# Shared mechanisms across the major psychiatric and neurodegenerative diseases

**DOI:** 10.1038/s41467-022-31873-5

**Published:** 2022-07-26

**Authors:** Thomas S. Wingo, Yue Liu, Ekaterina S. Gerasimov, Selina M. Vattathil, Meghan E. Wynne, Jiaqi Liu, Adriana Lori, Victor Faundez, David A. Bennett, Nicholas T. Seyfried, Allan I. Levey, Aliza P. Wingo

**Affiliations:** 1 Goizueta Alzheimer’s Disease Center, Emory University School of Medicine, Atlanta, GA USA; 2 Department of Neurology, Emory University School of Medicine, Atlanta, GA USA; 3 Department of Human Genetics, Emory University School of Medicine, Atlanta, GA USA; 4 Department of Cell Biology, Emory University School of Medicine, Atlanta, GA USA; 5 Department of Psychiatry, Emory University School of Medicine, Atlanta, GA USA; 6 Rush Alzheimer’s Disease Center, Rush University Medical Center, Chicago, IL USA; 7 Department of Biochemistry, Emory University School of Medicine, Atlanta, GA USA; 8 Veterans Affairs Atlanta Health Care System, Decatur, GA USA

**Keywords:** Depression, Post-traumatic stress disorder, Alzheimer's disease

## Abstract

Several common psychiatric and neurodegenerative diseases share epidemiologic risk; however, whether they share pathophysiology is unclear and is the focus of our investigation. Using 25 GWAS results and LD score regression, we find eight significant genetic correlations between psychiatric and neurodegenerative diseases. We integrate the GWAS results with human brain transcriptomes (*n* = 888) and proteomes (*n* = 722) to identify *cis*- and *trans*- transcripts and proteins that are consistent with a pleiotropic or causal role in each disease, referred to as causal proteins for brevity. Within each disease group, we find many distinct and shared causal proteins. Remarkably, 30% (13 of 42) of the neurodegenerative disease causal proteins are shared with psychiatric disorders. Furthermore, we find 2.6-fold more protein-protein interactions among the psychiatric and neurodegenerative causal proteins than expected by chance. Together, our findings suggest these psychiatric and neurodegenerative diseases have shared genetic and molecular pathophysiology, which has important ramifications for early treatment and therapeutic development.

## Abstract (web summary)

Studying the shared genetic etiology of disease can help improve diagnosis and treatment. Here, the authors find evidence for shared genetic and molecular pathophysiology between several common psychiatric and neurodegenerative diseases using results of 25 GWAS and large-scale human brain transcriptomic and proteomic sequencing.

## Introduction

Several lines of evidence link psychiatric disorders that typically manifest in early-adulthood or mid-adulthood with late-life neurodegenerative diseases. These psychiatric disorders include major depressive disorder, bipolar disorder, schizophrenia, anxiety disorders, post-traumatic stress disorder, problematic alcohol use, and the neurodegenerative diseases include Alzheimer’s disease, Lewy body dementia, Parkinson’s disease, amyotrophic lateral sclerosis, and frontotemporal dementia. First, individuals experiencing one of these psychiatric conditions have up to four times higher risk for developing dementia or a neurodegenerative disease later in life^1–6^. Second, about 65% of people affected by a neurodegenerative disease experience debilitating psychiatric symptoms during course of the illness^7^. Third, recent evidence suggests a shared genetic risk between schizophrenia and Parkinson’s disease^8,9^. Given these connections, we hypothesized that there is a shared genetic and molecular basis among these psychiatric and neurodegenerative diseases.

Insights into mechanisms shared among these psychiatric and neurodegenerative diseases could fuel the development of novel, effective therapeutics for *both* the psychiatric and neurodegenerative diseases and for the psychiatric symptoms that arise during the course of the neurodegenerative diseases. Moreover, because many neurodegenerative diseases have an incubation period of a decade or more prior to symptoms^10,11^, treatments for early- or mid-adulthood psychiatric disorders that target the shared mechanisms may mitigate risk for dementia and neurodegeneration later in life. Given the current pressing need for more effective treatments for both the psychiatric and neurodegenerative conditions, therapeutics that target their shared mechanisms may have far-reaching utility^12–16^.

Brain proteins are promising targets for drug discovery for these brain illnesses. Proteins are generally stable and often final executors of cellular processes or biological functions and constitute the vast majority of current medication targets^17^. Moreover, abnormal protein accumulation, conformations, and interactions are common features of neurodegenerative diseases^18^. Studying brain proteins directly rather than transcripts avoids the complication that brain mRNAs levels are generally not well correlated with brain protein abundance, likely due to several layers of post-transcriptional, translational, and post-translational regulation^19–24^. Lastly, proteins that function in the same biological pathway often physically interact, which may be harnessed to infer shared pathophysiology.

Here, we test the hypothesis that certain psychiatric and neurodegenerative diseases have a shared genetic and molecular basis using two approaches. First, we performed a large-scale pairwise genetic correlation among the aforementioned psychiatric and neurodegenerative diseases using LD score regression^25^ and the latest genome-wide association study (GWAS) results. We also included brain structural variation to help gauge these correlations since brain structural changes in certain regions may either predispose to illness or result from the illness (e.g., hippocampus and Alzheimer’s disease). These genetic correlations represent a genome-wide level of correlation. Second, we sought to identify specific genes that confer disease risk through their effects on brain protein abundance through integrating each GWAS results with the largest set of deeply profiled human brain proteomes (*n* = 722) using multiple complementary approaches – proteome-wide association study (PWAS), Mendelian randomization, and genetic colocalization analysis^26–28^. The three analytical approaches applied in a stepwise fashion enabled the identification of proteins that are consistent with pleiotropy or a causal role in each disease (and will be referred to as consistent with a causal role or causal proteins for simplicity henceforth). For proteins implicated in the pathogenesis of the investigated psychiatric and neurodegenerative conditions, we determined shared and distinct causal proteins and examined for evidence of enrichment of physical protein-protein interactions, which would be expected for proteins involved in the same molecular or biological pathways. We then applied the same analytical pipeline to 888 human brain transcriptomes to present another layer of evidence. Our findings implicated several distinct and shared causal brain proteins and elucidated potential shared mechanisms among these psychiatric and neurodegenerative diseases, paving the way for precision medicine and therapeutic development.

## Results

### Study design

**Fig. 1:**
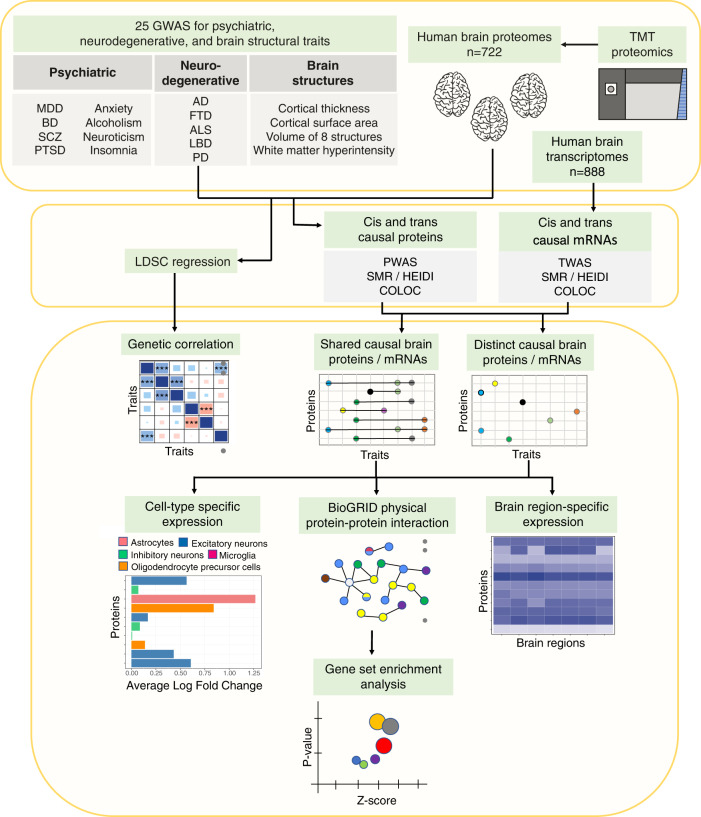
Study design. This is a summary of the analyses performed in this study.

We leveraged the latest GWAS findings for eight psychiatric traits (major depressive disorder (MDD)^29^, bipolar disorder (BD)^30^, schizophrenia^31^, anxiety^31^, post-traumatic stress disorder (PTSD)^32^, alcoholism^33^, neuroticism^34^, and insomnia^35^), five neurodegenerative diseases (Alzheimer’s disease (AD)^36,37^, Lewy body dementia (LBD)^38^, frontotemporal dementia (FTD)^38^, amyotrophic lateral sclerosis (ALS)^39^, and Parkinson’s disease (PD)^40^), and 11 brain structural endophenotypes (white matter hyperintensity^41^ (WMH), cortical thickness^42^, cortical surface area^42^, and volume of the hippocampus^43^, amygdala, nucleus accumbens, caudate nucleus, putamen, globus pallidus, thalamus, and brainstem^44^, Fig. 1, Table 1). We included two GWAS of AD^36,37^ because they differed in study design. One focused only on clinically diagnosed AD while the other also included a substantial proportion of individuals from the UK Biobank using family history of dementia as proxy-cases or controls. From these GWAS results, we first examined pairwise genetic correlations among these brain traits through LD score regression to determine whether there is a shared genetic basis among these psychiatric and neurodegenerative diseases. Next, we identified *cis-* and *trans*-regulated brain proteins that are consistent with a causal role in each trait by integrating 722 deep human brain proteomes (Table S1) with result of each GWAS using a combination of PWAS^26^, summary data-based Mendelian randomization (SMR)^27^, and colocalization analysis^28^. For brain proteins with evidence consistent with a causal role within and among the groups of traits, we examined brain expression by region and cell type and tested for evidence for protein-protein interactions (PPI). Lastly, we gleaned biological processes from these shared causal proteins and PPI networks to identify shared mechanisms between the investigated psychiatric and neurodegenerative diseases.

**Table 1:** Summary of the source GWAS and number of causal proteins for each brain trait identified through PWAS, SMR/HEIDI, and COLOC

	GWAS statistics		Causal protein counts			Causal proteins shared among traits		
	*N*	significant SNPs	*cis*	*trans*	total	Psychiatric	Neuro-degenerative	Brain structure
**Psychiatric**								
Major depressive disorder	807,553	9744	96	2	98	46%	2%	8%
Bipolar disorder	51,710	240	70	0	70	44%	3%	7%
Schizophrenia	77,096	14,724	156	2	158	31%	3%	7%
Anxiety	175,163	166	4	0	4	75%	50%	25%
Post-traumatic stress disorder	214,408	4607	16	0	16	44%	6%	13%
Alcoholism	300,789	1857	19	1	20	45%	10%	5%
Neuroticism	390,278	7759	122	2	124	34%	4%	6%
Insomnia	386,533	463	29	0	29	41%	0%	10%
**Neurodegenerative**								
Alzheimer’s disease (AD1)	455,258	2357	16	2	18	39%	22%	6%
Alzheimer’s disease (AD2)	63,926	1506	3	1	4	50%	50%	0%
Frontotemporal dementia	12,908	29	0	0	0	–	–	–
Amyotrophic lateral sclerosis	80,610	182	6	0	6	17%	0%	0%
Lewy body dementia	6618	189	0	0	0	–	–	–
Parkinson’s disease	482,730	3464	19	1	20	25%	10%	15%
**Brain structure**								
Hippocampal volume	26,814	461	2	0	2	0%	0%	50%
Nucleus accumbens volume	32,562	138	3	0	3	0%	0%	0%
Amygdala volume	34,431	0	0	0	0	–	–	–
Putamen volume	37,571	1465	6	0	6	50%	17%	50%
Brainstem volume	28,809	1063	10	0	10	50%	10%	30%
Caudate nucleus volume	37,741	141	7	0	7	14%	0%	29%
Globus pallidus volume	34,413	272	7	0	7	14%	14%	43%
Thalamus volume	34,464	50	2	0	2	50%	0%	50%
Cortical thickness	33,992	370	19	0	19	37%	5%	21%
White matter hyperintensity	48,454	1340	19	1	20	30%	0%	15%
Cortical surface area	33,992	1127	20	0	20	40%	15%	15%

The sample size (N) for each trait is the number of samples that contributed to the GWAS results used in our analysis, which may be smaller than the sample reported in the original GWAS publication due to data access limitations or restricting to samples of European ancestry. GWAS-significant SNPs were defined as SNPs with *p* value <5 × 10^−8^. A protein is considered shared with another trait group if it is also causal for any other trait in that group. Since different traits have different number of identified causal proteins, we examined the percentage of the causal proteins of each trait that is shared within a trait group or between two trait groups. This information is also depicted in Figs. 3A and 4A.

### Genetic correlations *within* and *between* the psychiatric, neurodegenerative, and brain structural groups

**Fig. 2:**
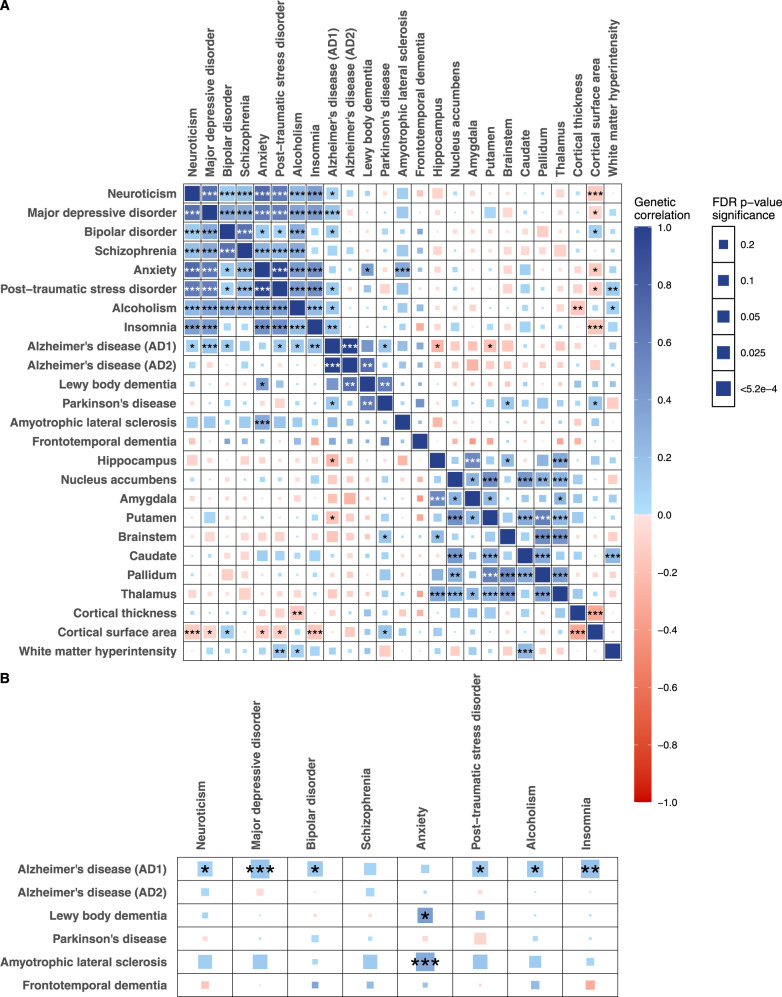
Genetic correlations across brain structures, psychiatric, and neurodegenerative diseases. **A** Pairwise genetic correlations among the 24 brain traits using LD score regression. **B** Genetic correlations between the psychiatric and neurodegenerative traits based on LD score regression. The color denotes the magnitude and direction of correlation. The size of the colored square reflects the magnitude of the *p* value. The asterisk indicates FDR *p* value: * FDR *p* < 0.05; ** FDR *p* < 0.01, *** FDR *p* < 0.001. The exact *p* values are provided in Table S2.

To estimate genetic correlations between traits within and between the groups of psychiatric, neurodegenerative, and brain structural phenotypes, we performed LD score regression^25^ using the GWAS summary statistics from participants of European descent (Fig. 2A, Table S2). The LD score regression approach, by design, is robust to sample overlap in the GWAS and minimizes potential bias by differences in LD structure^25^. We found many significant positive genetic correlations among the psychiatric disorders, consistent with prior studies^45–47^, and among the brain structures, also consistent with a prior study^42^. Notably, we found positive pairwise genetic correlations between AD, PD, and LBD, respectively, suggesting a shared genetic susceptibility among these three common neurodegenerative diseases (at FDR *p* < 0.05, Fig. 2A, Table S2).

Between the neurodegenerative and brain volumetric phenotypes (hippocampus, putamen, brainstem, cortical surface area), we found evidence of significant genetic correlations (FDR *p* < 0.05, Fig. 2A, Table S2). Between the psychiatric and brain structural phenotypes (cortical surface area, cortical thickness, and WMH), we also found significant genetic correlations (FDR *p* < 0.05, Fig. 2A, Table S2). Remarkably, we found positive genetic correlations among the neurodegenerative and psychiatric traits—between AD and neuroticism, MDD, BD, PTSD, alcoholism, and insomnia, respectively; between LBD and anxiety symptoms; and between ALS and anxiety symptoms (FDR *p* < 0.05, Fig. 2B, Table S2). These novel findings are consistent with the hypothesis that the psychiatric disorders share a common genetic basis with neurodegenerative diseases.

### *cis-*regulated proteins consistent with a causal role in 24 brain traits

To further expand on the evidence of a shared genetic basis among the psychiatric and neurodegenerative diseases, we sought to identify *specific* genes that confer disease risk through their effects on brain protein abundance. To this end, we performed a PWAS of each brain trait to identify *cis*-regulated proteins associated with each trait using 722 reference human brain proteomes. The brain proteomes were profiled mostly from the frontal cortex using tandem mass tag mass spectrometry (Table S1). Prior to the integration, in each of the proteomic datasets separately, we performed quality control, normalization, removal of effects of clinical characteristics and technical factors, and standardizing the protein abundance using Z-scale. Then we merged these proteomic datasets into a combined proteomic profile and performed surrogate variable analysis. We regressed out effects of the identified hidden confounding factors before applying the integration with GWAS results. After quality control and normalization, 9363 proteins remained in the combined proteomic profile, and of these, 2909 had significant SNP-based heritability to be included in the PWAS. The effect of genetic variants on protein abundance for each of these 2909 proteins were estimated using variants within a 500Kb window around the gene. On average, 11.8% of the variance in measured protein abundance (median: 6.9%, range [0.04–68.1%]) was attributable to *cis* genetic variation (Table S3). Subsequently, the PWAS for each trait was performed using FUSION^26^, which integrates genetic effect on protein abundance with genetic effect on a given trait. Significant proteins for each trait from the PWAS after FDR adjustment are listed in Table S4. In total, we performed 25 independent PWAS that collectively identified 839 *cis*-regulated proteins associated with one of the 24 brain traits.

Next, we tested whether the *cis*-regulated proteins identified in each PWAS mediate the association between the genetic variants and disease using Mendelian randomization following the SMR pipeline^27^. Since association between genetic variants, protein, and disease can be due to linkage disequilibrium, pleiotropy, or causality, we also tested for the probability of linkage disequilibrium versus pleiotropy or causality using the heterogeneity in dependent instrument test (HEIDI)^27^ and retained only associations due to pleiotropy or causality. Additionally, we used a complementary Bayesian method, COLOC^28^, to examine colocalization of the disease-associated variants and the protein abundance-associated variants.

After considering results from the PWAS, SMR/HEIDI, and COLOC, we found 651 *cis*-regulated proteins consistent with causality or pleiotropy for one or more of the 24 traits and listed the number of these proteins for each trait in Table 1 and the specific proteins for each trait in Table S4. For simplicity, we will refer to the *cis*-regulated proteins consistent with causality or pleiotropy as *cis* causal proteins henceforth.

### *trans-*regulated proteins consistent with a causal role in 24 brain traits

Beyond *cis*-regulated proteins, we sought to identify *trans*-regulated proteins that are consistent with a causal role in each of these brain traits. We first performed a *trans*-pQTL analysis among the genome-wide significant SNPs identified by the 25 GWAS using the 722 reference human brain proteomes and corresponding genome-wide genotypes. *Trans*-pQTLs were declared for SNPs associated with protein abundance at *p* < 5 × 10^−8^ and are outside of the 500 kb window of the protein-coding gene. We found *trans*-pQTLs for 13 proteins among these GWAS-significant SNPs. Subsequently, we performed Mendelian randomization using the *trans*-SMR/HEIDI pipeline^27^ to identify the *trans* proteins that mediate the association between the GWAS-significant SNPs and disease through pleiotropy or causality. We identified 12 *trans*-regulated proteins consistent with causality or pleiotropy for several of these brain traits (Table 1, Table S4). For simplicity, we will refer to the *trans*-regulated proteins consistent with causality or pleiotropy as *trans* causal proteins henceforth.

### Shared causal proteins within the trait groups

**Fig. 3:**
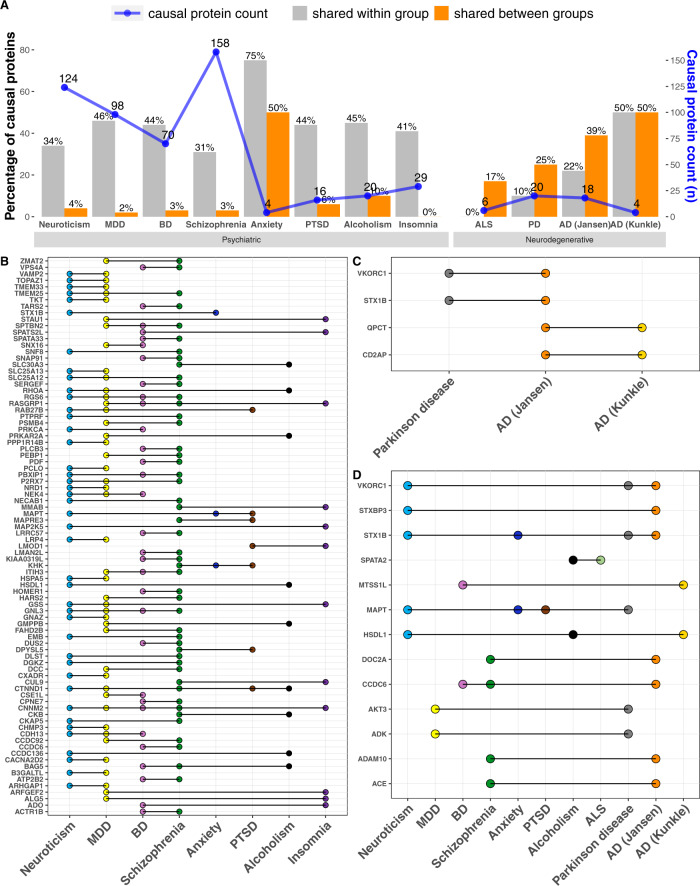
Causal brain proteins shared within and between groups of psychiatric and neurodegenerative diseases. **A** Number of causal proteins and percent shared *within* group and *between* groups of psychiatric and neurodegenerative diseases. **B** Causal proteins shared within the psychiatric group. **C** Causal proteins shared within the neurodegenerative disease group. **D** Causal proteins shared between the psychiatric and neurodegenerative groups. Anxiety, PTSD, and insomnia are not listed because they do not have shared causal proteins with the neurodegenerative diseases.

**Fig. 4:**
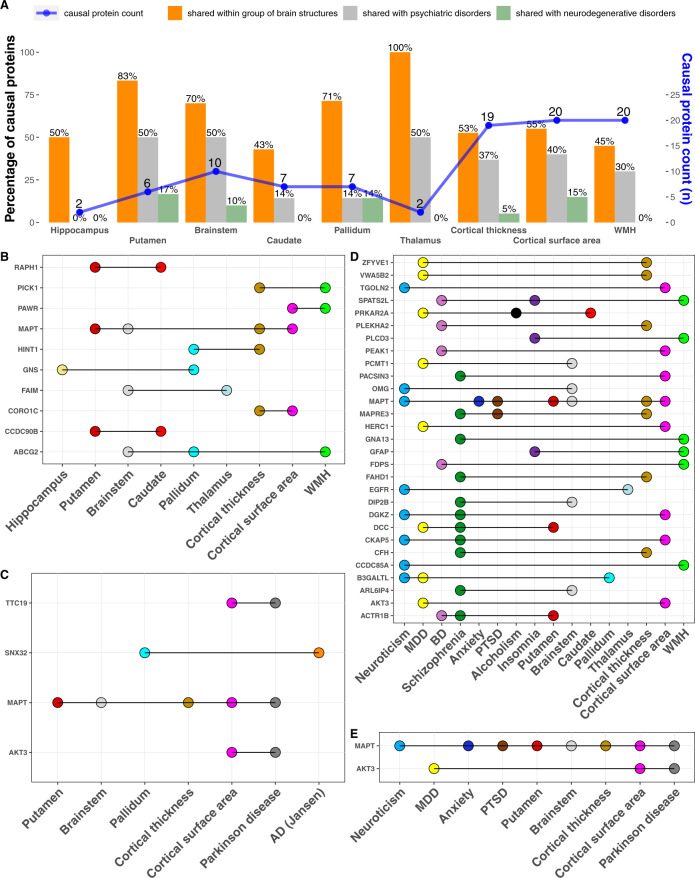
Causal proteins shared *within* the brain structure traits and shared *between* the brain structure group, the psychiatric group, and neurodegenerative group, respectively. **A** Number of causal proteins and percent shared *within* the brain structure group and *between* the brain structure group and the psychiatric and neurodegenerative group, respectively. **B** Causal proteins shared within brain structures. **C** Causal proteins shared between brain structures and neurodegenerative diseases. **D** Causal proteins shared between brain structures and psychiatric traits. **E** Causal proteins shared among the three groups.

After elucidating the *cis* and *trans* causal proteins for each of the brain traits, we next identified proteins that are mutually causal between traits within the psychiatric, neurodegenerative, and brain structural categories, respectively. Since different traits have a different number of identified causal proteins, we examined the percentage of the causal proteins of each trait that is shared within a group. Overall, we observed a substantial percentage of shared causal proteins between pairs of traits within each group—an average of 45% of sharing within the psychiatric group (range [31–75%]), 21% of sharing within the neurodegenerative group (range [0–50%]), and 30% of sharing within the brain structure group (30% [0% to 50%], Figs. 3A and 4A; Table 1). Individual proteins shared between pairs of traits are listed in Table S5.

Within the psychiatric group, many causal proteins were shared among three or more traits (Fig. 3B). Specifically, 14 proteins were shared among three psychiatric traits, three proteins (GNL3, RASGRP1, RGS6) were shared among four psychiatric traits, and two proteins (CTNND1, CNNM2) were shared among five psychiatric traits (Fig. 3B, Table S5). Within the neurodegenerative group, two proteins (VKORC1 and STX1B) were shared between AD and PD, and two other proteins (QPCT and CD2AP) were shared between the clinical diagnosis of AD and AD based on both clinical diagnosis and family history of dementia (Fig. 3C, Table S5). Within the brain morphology group, MAPT is mutually causal in four traits and ABCG2 mutually causal in three traits (Fig. 4B, Table S5). Together, these findings highlight the shared genetic predisposition within the group of psychiatric, neurodegenerative, and brain structure traits.

### Shared causal proteins between the trait groups

Between the psychiatric and neurodegenerative groups, we found 13 shared causal proteins, which is 30% of the total 44 identified causal proteins for the neurodegenerative diseases (Fig. 3D, Table S5). This striking percentage of shared causal proteins is supportive of the hypothesis of a shared genetic and molecular basis between the psychiatric and neurodegenerative groups.

Among these 13 shared causal proteins, eight were shared between a pair of traits (a psychiatric and neurodegenerative disease), three were shared among three traits (a psychiatric, neurodegenerative, and either another psychiatric or neurodegenerative disease), and two were shared among four traits (multiple psychiatric and neurodegenerative diseases; Fig. 3D). Additionally, we found a positive relationship between the degrees of genetic correlation and percentages of shared casual proteins among the psychiatric and neurodegenerative diseases (Spearman rho = 0.39; *p* = 0.01; Fig. S1).

Between the neurodegenerative and brain structural groups, there were four shared causal proteins, with MAPT being shared among PD, putamen volume, brainstem volume, cortical thickness, and cortical surface area (Fig. 4C, Table S5). Between the psychiatric and brain structural categories, there were 29 shared causal proteins, with MAPT shared among three psychiatric traits and four brain structures (Fig. 4D). Notably, AKT3 and MAPT were shared among multiple traits that belong to all three groups of psychiatric, neurodegenerative, and brain structures (Fig. 4E). Together, these shared causal proteins underscore a shared genetic susceptibility among the psychiatric and neurodegenerative diseases.

### Brain-region and cell-type expression of the causal proteins

**Fig. 5:**
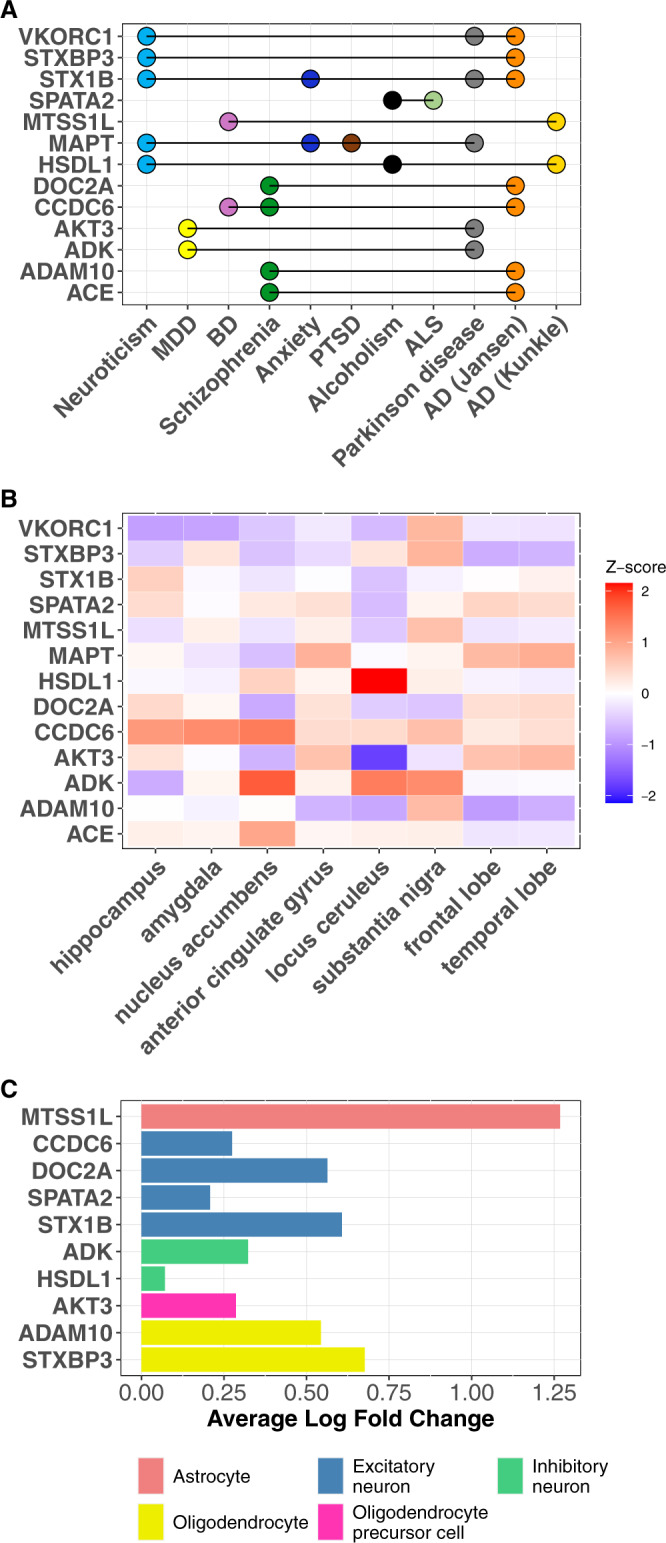
Expression in different brain regions and cell types for the 13 causal proteins shared between the psychiatric and neurodegenerative diseases. **A** List of the 13 proteins mutually causal in both the psychiatric and neurodegenerative diseases. **B** Expression of the 13 shared causal proteins in different brain regions based on human brain gene expression data from the Allen Brain Atlas. Higher Z score (red) indicates higher expression, lower Z score (blue) indicates lower expression in that brain region. **C** Expression of the 13 shared causal proteins in different cell types based on human single-cell RNA-sequencing data. Whether the gene expression in one particular cell-type differed from its expression in all the other four cell types was determined using the Wilcoxon rank sum test. Significant difference was defined as those with Bonferroni-adjusted *p* value < 0.05 (adjusted for all 17,775 genes). Only 10 of 13 genes were highly expressed in a particular cell type and depicted here. The other 3 genes were expressed similarly across the different cell types.

We examined the expression of the 13 causal proteins shared between the psychiatric and neurodegenerative diseases in different brain regions that are relevant to these conditions, including the amygdala, nucleus accumbens, anterior cingulate gyrus, frontal cortex, hippocampus, locus coeruleus, substantia nigra, and temporal lobe. To compare their expression across the different brain regions, we examined the Z-score normalized human brain expression data from the Allen Brain Atlas^48^ (Fig. 5A, B; Table S6). We noted that the expression level of each gene varies across these brain regions and the pattern of relative gene expression differs across genes. For instance, HSDL1 expression is much higher in the locus coeruleus than in the other brain regions, while AKT3 expression is much lower in the locus coeruleus than in the other brain regions. These observations may be useful for informing the design of follow-up mechanistic studies.

We also examined expression of these 13 shared causal proteins in GABAergic, glutamatergic, and non-neuronal cells using mouse single-cell RNA-sequencing data from the isocortex and hippocampal formation^49^. We found that these 13 proteins had different anatomical expression patterns in the brain (Fig. S2).

To understand gene activities across the brain cell-types, we examined brain cell-type specific expression of these 651 causal proteins using human single-cell RNA sequencing data profiled from the dPFC of cognitively normal donors^50^. Among the 13 shared causal proteins, 10 had higher expression in one brain cell type compared to the rest of the other brain cell types (Fig. 5C, Table S7). Notably, four of these proteins were highly expressed in excitatory neurons (CCDC6, DOC2A, SPATA2, STX1B), two were highly expressed in inhibitory neurons (ADK, HSDL1), and two were highly expressed in oligodendrocytes (ADAM10, STXBP3) (Fig. 5C, Table S7). Cell-type expression of all distinct and shared causal proteins for these 24 psychiatric, neurodegenerative, and brain structural traits are presented in Table S7.

### Protein-protein interactions between the psychiatric and neurodegenerative causal proteins

**Fig. 6:**
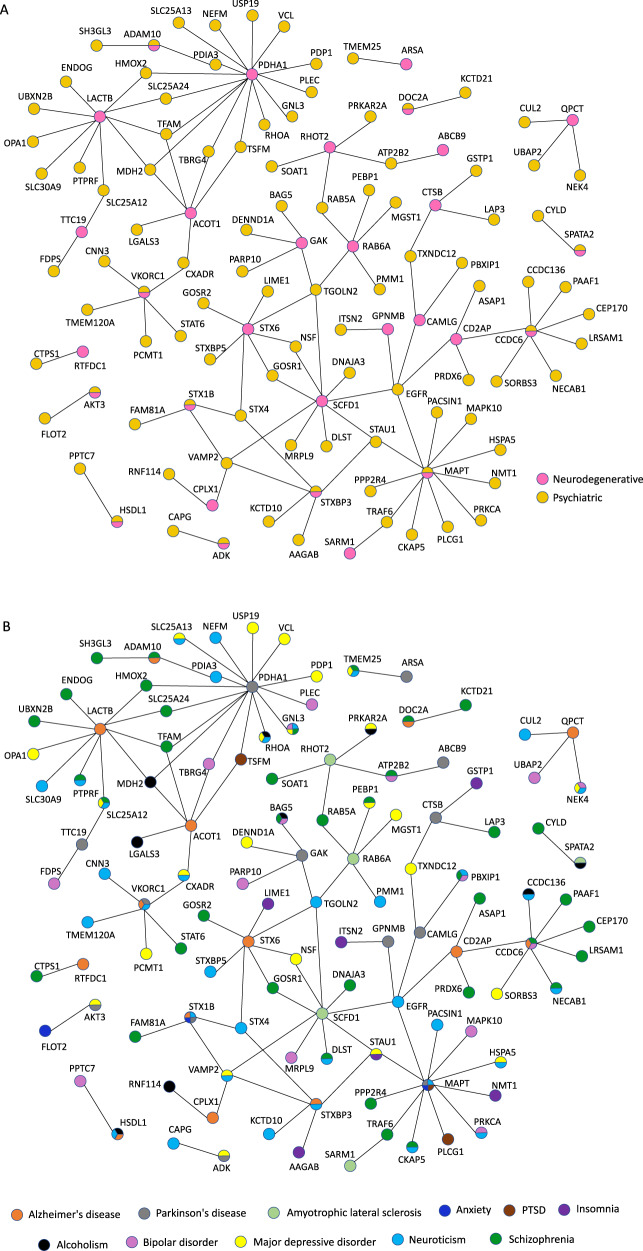
Physical protein-protein interactions (PPIs) between the psychiatric causal proteins and the neurodegenerative causal proteins. **A** PPIs between the psychiatric causal proteins (colored yellow) and neurodegenerative causal proteins (colored pink). We only considered physical PPI between a psychiatric and a neurodegenerative causal protein (and not between a psychiatric and another psychiatric causal protein or between a neurodegenerative and another neurodegenerative causal protein). There is a total of 118 psychiatric and neurodegenerative causal proteins that have physical PPIs with one another. See complete results in Table S8. **B** PPIs between the psychiatric causal proteins and neurodegenerative causal proteins. The same information in A is presented but each brain trait is depicted with a distinct color. See complete results in Table S8.

Thus far, we have considered proteins that are mutually causal in the psychiatric and neurodegenerative diseases as evidence for shared susceptibility. Another way shared mechanism may arise is through protein-protein interaction (PPI) between causal proteins. We hypothesized that there is a higher level of PPI between the psychiatric and neurodegenerative causal proteins than by chance alone. To test this, we determined the number of PPIs between a psychiatric causal protein (407 total proteins) and a neurodegenerative causal protein (44 total proteins) using physical PPI data from BioGRID^51^. Physical PPIs in BioGRID were curated from primary experimental evidence in peer-reviewed biomedical literature. We found evidence for 120 PPIs between 99 psychiatric and 30 neurodegenerative causal proteins (of which eleven are shared between the two groups). Consistent with our hypothesis, this reflects 2.6-folds more physical PPIs between the causal psychiatric and causal neurodegenerative proteins than expected by chance alone (bootstrap *p* = 0.003; Fig. 6; Table S8). These PPIs were among 118 psychiatric and neurodegenerative causal proteins. These 118 shared and interacting causal proteins may provide a window into the shared mechanisms between the psychiatric and neurodegenerative disease groups.

Among these 118 shared and interacting causal proteins, there are a few notable proteins with wide-spread interactions with other causal proteins. For example, PDHA1 (a PD causal protein) physically interacts with 15 psychiatric causal proteins; MAPT (another PD causal protein) physically interacts with 11 psychiatric causal proteins; LACTB (an AD causal protein) interacts with 10 psychiatric causal proteins; SCFD1 (an ALS causal protein) interacts with 9 psychiatric causal proteins; and CCDC6 and STX6 (AD causal proteins) each interacts with 7 psychiatric causal proteins, respectively (Fig. 6A, B; Table S8). These interactions provide important evidence supporting the hypothesis of shared molecular mechanisms between the psychiatric and neurodegenerative disease groups.

### Shared biological processes between the psychiatric and neurodegenerative diseases

**Fig. 7:**
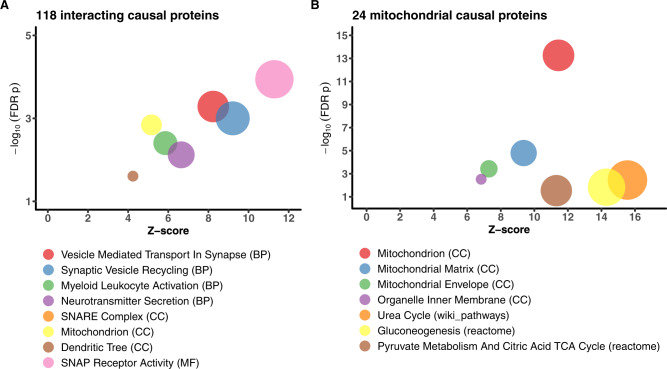
Gene set enrichment analysis (GSEA) for the 118 shared and interacting psychiatric and neurodegenerative causal proteins. **A** Biological processes that the 118 shared and interacting causal proteins were enriched in. The plot depicts only selected processes. The full results are presented in Table S9,b. **B** Biological functions that the 24 mitochondrial proteins were enriched in. These 24 mitochondrial proteins are a subset of the 118 shared and interacting causal proteins.

To gain insights into shared molecular processes, we performed gene set enrichment analysis on the 13 shared causal proteins and the 118 shared and interacting causal proteins, respectively, using Gene Ontology, KEGG, Wiki pathways, CORUM, and REACTOME databases. The 13 causal proteins shared between the psychiatric and neurodegenerative groups were enriched for calcium ion-regulated exocytosis and for localization of proteins to the synapse (FDR *p* < 0.05, Table S9, a). The 118-interacting psychiatric and neurodegenerative causal proteins were enriched for several processes—neurotransmitter secretion, vesicle-mediated transport in synapse, synaptic vesicle recycling, SNAP receptor activity, myeloid leukocyte activation, and mitochondrial processes among others (at FDR *p* < 0.05, Fig. 7A; Table S9, b).

To follow-up on these findings, we next examined PPI and gene set enrichment among these 118 interacting causal proteins using specialized human brain synaptic and mitochondrial databases, respectively. We used a curated human synaptic proteome database^52^ that included 7907 pre-synaptic, synaptic, and post-synaptic proteins. We found 102 synaptic proteins among these 118 interacting causal proteins, which is a 2.2-fold enrichment for synaptic proteins (*p* < 9.5 × 10^−27^). Moreover, these 102 synaptic proteins were enriched for similar biological processes as those of the 118 proteins (FDR *p* < 0.05, Table S9,c).

To further investigate the enrichment of mitochondrial processes, we used MitoCarta3.0^53^, a curated database of 1137 human mitochondrion-related genes. Among the 118 interacting causal proteins, we identified 24 mitochondrial proteins, which is 3.7-fold enrichment of mitochondrial proteins (*p* < 2.3 × 10^−8^). Gene set enrichment analysis found that these 24 mitochondrial proteins were similarly distributed across the mitochondrial sub-compartments such as the matrix and the inner mitochondrial membrane (FDR *p* < 0.05; Fig. 7B; Table S9, d). We then queried the high-density human mitochondrial proximity interaction network^54^ and found an interactome of 66 proteins among these 24 mitochondrial proteins (Fig. S3).

Together, the results from using the general and specialized databases of curated synaptic and mitochondrial proteins implicate synaptic transmission and immune function as important processes in the shared mechanisms between the psychiatric and neurodegenerative diseases.

### Shared mechanisms at the brain mRNA level

To follow up on the brain protein findings, we applied the same analysis pipeline using 888 human brain transcriptomes profiled mostly from the frontal cortex. For simplicity, we will refer to mRNAs that are consistent with a causal role or pleiotropy as causal mRNAs. We identified unique and shared *cis-* and *trans-*causal mRNAs for each of the 24 brain traits (Table 2; S10–S12). There were 615 causal mRNAs for the psychiatric traits and 64 causal mRNAs for the neurodegenerative diseases. Consistent with our findings at the protein-level, we found shared causal mRNAs *within* each group and *between* the groups of psychiatric, neurodegenerative, and brain structural traits (Table 2; Table S12). Specifically, there were 24 shared causal mRNAs between the psychiatric and neurodegenerative diseases, which is 37.5% of the total identified neurodegenerative causal mRNAs (Table S13). Similar to our findings at the protein-level, there were 171 physical PPIs between the 145 psychiatric and neurodegenerative causal mRNAs (Table S14). This is 2.0 times more PPI than by chance alone (*p* = 0.008). Gene set enrichment analysis found that these 145 shared and interacting causal mRNAs were enriched for mitochondrial organization, transport, and fission; epigenetic regulation of gene expression; gene silencing; chromatin modifying enzymes; histone methyltransferase complex; RNA binding; metabolism of RNA; regulation of protein targeting; and protein ubiquitination (Table S15).

**Table 2:** Summary of the source GWAS and number of causal mRNAs identified through TWAS, SMR/HEIDI, and COLOC

	GWAS statistics		Causal mRNA counts			Causal mRNAs shared among traits		
	*N*	significant SNPs	*cis*	*trans*	total	Psychiatric	Neuro-degenerative	Brain structure
**Psychiatric**								
Major depressive disorder	807,553	9744	160	11	171	37%	2%	4%
Bipolar disorder	51,710	240	88	0	88	47%	1%	2%
Schizophrenia	77,096	14,724	243	5	248	25%	3%	4%
Anxiety	175,163	166	11	0	11	82%	46%	27%
Post-traumatic stress disorder	214,408	4607	37	0	37	43%	8%	11%
Alcoholism	300,789	1857	23	0	23	48%	26%	13%
Neuroticism	390,278	7759	154	5	159	40%	8%	8%
Insomnia	386,533	463	30	0	30	30%	13%	7%
**Neurodegenerative**								
Alzheimer’s disease (AD1)	455,258	2357	28	0	28	46%	25%	4%
Alzheimer’s disease (AD2)	63,926	1506	12	2	14	36%	29%	0%
Frontotemporal dementia	12,908	29	0	0	0	–	–	–
Amyotrophic lateral sclerosis	80,610	182	4	0	4	25%	0%	0%
Lewy body dementia	6,618	189	0	0	0	–	–	–
Parkinson’s disease	482,730	3464	25	0	25	32%	12%	28%
**Brain structure**								
Hippocampal volume	26,814	461	0	0	0	–	–	–
Nucleus accumbens volume	32,562	138	0	0	0	–	–	–
Amygdala volume	34,431	0	0	0	0	–	–	–
Putamen volume	37,571	1465	9	0	9	33%	22%	44%
Brainstem volume	28,809	1063	20	0	20	30%	10%	10%
Caudate nucleus volume	37,741	141	15	0	15	20%	0%	27%
Globus pallidus volume	34,413	272	12	0	12	8%	0%	42%
Thalamus volume	34,464	50	3	0	3	0%	0%	0%
Cortical thickness	33,992	370	10	0	10	40%	40%	30%
White matter hyperintensity	48,454	1340	29	0	29	31%	10%	14%
Cortical surface area	33,992	1127	29	0	29	35%	17%	14%

The sample size (N) for each trait is the number of samples that contributed to the GWAS results used in our analysis, which may be smaller than the sample reported in the original GWAS publication due to data access limitations or restricting to samples of European ancestry. GWAS-significant SNPs were defined as SNPs with *p* value < 5 × 10^−8^. A mRNA is considered shared within a trait group if it is also causal for any other trait in that group. Since different traits have different number of identified causal mRNAs, we examined the percentage of the causal mRNAs of each trait that is shared within a trait group or between two trait groups.

The PWAS included 2909 heritable proteins and the TWAS included 6735 heritable mRNAs, of which 1726 genes were common between these proteins and mRNAs. We found that, on average, 39.6% of the causal mRNAs were also causal proteins, and 32.3% of the causal proteins were also causal mRNAs (Table S16). *ADAM10* and *CCDC6* were the shared causal genes for the psychiatric and neurodegenerative diseases at both the mRNA and protein levels.

## Discussion

In this study, we present robust and novel evidence for a shared genetic and molecular basis between the psychiatric illnesses that typically manifest in early adulthood or mid-life and the neurodegenerative diseases that typically manifest in late life. The shared genetic susceptibility is demonstrated by the significant genetic correlations between the psychiatric and neurodegenerative diseases and the 13 shared causal proteins. More evidence of a shared molecular basis is seen by a nearly three-folds more physical PPIs between a causal psychiatric and a causal neurodegenerative protein, which implicates specific networks of genes and molecular processes involved in both groups of diseases. Furthermore, we found that the SNARE complex, vesicular transport, synaptic transmission, immune function, and mitochondrial processes are implicated in the shared mechanisms and pinpoint the brain proteins involved in these biological processes for further mechanistic investigation.

This shared genetic and molecular processes have far-reaching implications for the management and therapeutic development for these common brain illnesses. For context, these psychiatric disorders affect approximately 30% of the population annually in early or midlife (age < 65)^55,56^ while the neurodegenerative diseases affect about 44 million people worldwide, mostly in late-life, with no effective treatments to slow or stop the underlying neurodegeneration^18^. Furthermore, about 65% of the individuals with neurodegenerative diseases experience psychiatric symptoms (also referred to as neuropsychiatric symptoms) that are associated with faster cognitive decline, greater functional impairment, higher caregiver burden, and earlier institutionalization, but there are no safe and effective treatments for these neuropsychiatric symptoms^57–60^. Both of these psychiatric and neurodegenerative disease groups are the leading causes of disability worldwide and effective treatments for these brain illnesses are pressingly needed^12–16^. Given the shared molecular basis, treatments that focus on the shared pathophysiology may be able to treat not only the early / midlife psychiatric disorders but also the common neuropsychiatric symptoms of late-life neurodegenerative disorders, as well as mitigating the late-life neurodegeneration and dementia risk.

Another notable finding of this study is the identification of 651 brain proteins consistent with a causal role in the 24 brain traits. Of the 651 brain proteins, 394 were unique to psychiatric illnesses, 31 unique to neurodegenerative diseases, and 13 shared between the psychiatric and neurodegenerative diseases. Together with the causal transcripts identified for these brain illnesses in this and prior studies^46,61–64^, these causal proteins provide mechanistic insights and promising targets for therapeutic development to treat these common and debilitating brain illnesses.

While evidence of the role of synaptic transmission in the pathophysiology of these psychiatric disorders is still emerging^29,65,66^, the role of synaptic transmission and SNARE complex in the pathophysiology of neurodegenerative diseases is more established^67,68^. Here, we extend the literature in demonstrating that synaptic transmission, particularly involving the SNARE complex and SNAP receptor, is important in the shared mechanisms among the psychiatric and neurodegenerative diseases. Moreover, synaptic transmission is regulated by mitochondria in the neurons, which generate ATP to power the neurotransmission process and modulate the presynaptic calcium level, which in turn determines the release of neurotransmitters^69^. Therefore, mitochondria are vital for the maintenance of synaptic transmission and function^69,70^. Notably, we found that mitochondrial processes are implicated in the shared pathophysiology in psychiatric and neurodegenerative disease. While this is novel, it is consistent with the observation that mitochondrial dysfunction occurs early in the progression to neurodegeneration and continues into the late stage of the diseases^71–74^ because the shared mechanisms are more likely to be early acting since psychiatric disorders have onset age in early adulthood or midlife while neurodegenerative diseases manifest in late-life.

mRNA and protein expression levels for the same genes in the brain have been found to have modest correlations^19,20,22–24^; therefore, we expected our findings would likely reflect this. Here we found that 32% of the causal proteins are also causal mRNAs, consistent with the extant literature.

Microglia has emerged as important in the pathogenesis of AD^37,75^. For instance, approximately 5% of the 400 genes inferred from the AD GWAS showed high microglial expression^36^. We note that 10% of the AD causal proteins we identified are highly expressed in microglia compared to bulk brain tissue - CTSH (8 folds higher) and PLXDC2 (53 folds higher)^75^. Furthermore, TREM2 is one of the AD causal mRNAs we identified and it is an innate immunity receptor expressed selectively in microglia (56 folds higher than in bulk brain tissue)^37,75^.

Among the 13 shared causal proteins between the psychiatric and neurodegenerative groups (Fig. 3D), five have been studied in human participants or mouse models in one or more of these brain illnesses. For instance, mutations of *ADAM10* led to AD pathology in transgenic mice^76^ and mRNA expression level of *ADAM10* in the brain was differentially expressed between schizophrenia patients versus controls^77^, supportive of our findings. In another instance, mRNA expression of *DOC2A* in the brain was differentially expressed in AD patients versus controls^73^ and in schizophrenia patients versus controls^78^, also supportive of our findings. Additionally, mRNA expression level of *CCDC6* in the hippocampus was differentially expressed between AD patients and controls^73^ but it has not been studied in bipolar disorder or schizophrenia. Furthermore, mRNA level of *MTSS1L* in the brain was differentially expressed between AD patients versus controls^79^ but it has not been studied in bipolar disorder. Lastly, *STX1B* mRNA level in the brain was differentially expressed between AD patients versus controls^73^ but it has not been studied in neuroticism or Parkinson’s disease. These published results lend credence to our findings but also highlight that the identified shared causal proteins are novel targets for mechanistic studies in model systems to advance our understanding of the shared mechanisms between the psychiatric and neurodegenerative diseases.

Our findings should be considered in the context of the study’s limitations. For instance, the power to detect causal brain proteins for LBD and FTD was limited by the power of their underlying GWAS. Along these lines, MAPT was found to be a causal protein for multiple psychiatric disorders and Parkinson’s disease but not for AD, which is classically associated with abnormal MAPT accumulation. We note that MAPT is only nominally significant in the PWAS of clinical AD (*p* = 0.027, FDR = 0.52), perhaps due to power limitation, and future larger GWAS may help resolve this. In addition, we could not accurately account for cell-type heterogeneity in the PWAS due to lack of availability of human brain single-cell proteomic data. Furthermore, with 722 deep human brain proteomes, we could identify 2909 heritable proteins for the PWAS and SMR. With larger number of human brain proteomes, we are likely to identify more heritable brain proteins. Despite this limitation, we presented here the largest and deepest set of human brain proteomes available to our knowledge. Lastly, the GWAS and human brain proteomes were from participants of European ancestry, potentially limiting the generalizability of our findings to individuals of other ethnicities.

Our study has several strengths. First, we used the largest set of deep human brain proteomes to maximize the number of heritable proteins to be included in the PWAS, enabling examination of more proteins. Second, we consider 24 brain traits, including structural brain traits that are often intermediary phenotypes in neurodegenerative disease. Third, we identified both *cis-* and *trans-*regulated causal proteins. Fourth, we examined both the brain proteins and mRNAs in our analyses. Lastly, the causal proteins identified here are highly promising for therapeutic development since causal genes derived from GWAS and Mendelian randomization have been shown to double the odds that a therapeutic target receives drug approval as a treatment of a human illness^17,80^.

In summary, we demonstrated here that the major psychiatric and neurodegenerative diseases have shared genetic susceptibility and pathophysiology and identified 13 shared causal proteins, 118 interacting causal proteins, and the central role of synaptic transmission, immune function, and mitochondrial processes in the shared pathogenesis. The genetic and molecular connection between these two groups of common brain illnesses have important implications for disease management and therapeutic development with regard to precision medicine, early treatment and prevention of neurodegeneration and dementia risk.

## Methods

### GWAS summary statistics

The GWAS summary association statistics were obtained from 25 studies that included eight psychiatric traits (MDD^29^, BD^30^, schizophrenia^31^, anxiety^31^, PTSD^81^, alcoholism^32^, neuroticism^34^ and insomnia^35^), five neurodegenerative diseases (AD^36,37^, LBD^38^, FTD^38^, ALS^39^, and PD^40^), and 11 brain structural endophenotypes (WMH^41^, cortical thickness^41^, cortical surface area^42^, and volume of the hippocampus^43^, amygdala, nucleus accumbens, caudate nucleus, putamen, globus pallidus, thalamus, and brainstem^44^). All studies were of people of European descent. We included two GWAS of AD^36,37^ because they differed in study design. One focused only on clinical diagnosis of AD^37^ while the other included clinical diagnosis of AD and AD-by-proxy using family history of dementia in participants from the UK Biobank^36^.

### Brain proteomics

#### Brain regions and cohorts

We analyzed data from the following brain regions per cohort: dorsolateral prefrontal cortex (dPFC) and frontal cortex for ROS/MAP, dPFC for Banner, and parahippocampal gyrus for MSBB (Table S1). Details for proteomic data generation procedures can be found in Wingo et al.^82^ (ROS/MAP dPFC), Wingo et al.^23^ (Banner dPFC), and Johnson et al.^24^ (ROS/MAP frontal cortex and MSBB samples) and are summarized below.

The Religious Orders Study (ROS) and Rush Memory and Aging Project (MAP), collectively referred to as ROS/MAP, are community-based cohort studies of cognitive decline, dementia, and aging^83^. ROS recruits older priests, monks, and nuns from throughout the United States, while MAP recruits people from greater Chicago-area assisted living facilities. All participants undergo annual cognitive and clinical assessments. All participants are organ donors, provide informed consent, and sign an Anatomical Gift Act and repository consent to allow their data and biospecimens to be repurposed. An Institutional Review Board of Rush University Medical Center approved the studies.

The Arizona Study of Aging and Neurodegenerative Disorders, conducted by Banner Sun Health Research Institute (Banner), primarily recruits cognitively normal volunteers from Phoenix, AZ area retirement communities, with some additional special recruitment of participants with AD or PD^84^. All participants receive annual standardized medical, cognitive, neurological, and movement assessments. Participants or their legal representatives sign an Institutional Review Board-approved consent form allowing for brain donation, use of donated biospecimens for approved future research, and genetic studies.

The MSBB brains were selected from the Mount Sinai/JJ Peters VA Medical Center Brain Bank^85^. The brains were selected such that they showed no non-AD neuropathology but otherwise span the full range of cognitive and neuropathologic disease severity.

#### Tissue homogenization and protein digestion

For ROS/MAP dPFC and Banner samples, tissue homogenization was performed as described previously^23,86^. Brain tissue samples (100 mg wet tissue weight each) were homogenized in 1.5 ml Rino tubes containing 500 ml lysis buffer (8 M urea, 10 mM Tris, 100 mM NaHPO4, pH 8.5), HALT protease and phosphatase inhibitor cocktail, and ~100 μl stainless steel beads (0.9–2.0 mm blend; NextAdvance) using a Bullet Blender (NextAdvance). Tissues were added immediately after excision and samples were blended for two 5-minute intervals at 4 C. Lysates were transferred to 1.5 ml Eppendorf Lobind tubes and sonicated at 30% amplitude for three 5 s cycles, with samples placed on ice for 15 s between each sonication cycle. Samples were subsequently centrifuged at 15,000 × *g* for five minutes. The supernatant was transferred to new tubes and protein concentration was measured using bicinchoninic assay (BCA). A 100 μg aliquot was taken from each sample, and the volumes were normalized with lysis buffer. An equal amount from each sample was aliquoted and digested in parallel to make up the global internal standard (GIS) that was included in each TMT batch. Samples were reduced with 1 mM dithiothreitol at room temperature for 30 min, then with 5 mM iodoacetamide alkylation in the dark for 30 min. Lysyl endopeptidase (Wako) at 1:100 (w/w) was added and samples underwent digestion overnight, followed by a sevenfold dilution with 50 mM ammonium bicarbonate. Trypsin (Promega) at 1:50 (w/w) was added and samples underwent another 16 h of digestion. Samples were acidified to a final concentration of 1% (vol/vol) formic acid (FA) and 0.1% (vol/vol) trifluoroacetic acid (TFA) and desalted with a 30-mg solid-phase extraction sorbent column (Oasis Hydrophilic-Lipophilic Balance). Each column was first rinsed with 1 ml of methanol, washed with 1 ml of 50% (vol/vol) acetonitrile (ACN) and equilibrated with 2 × 1 ml of 0.1% (vol/vol) TFA. The samples were then loaded and each column was washed with 2 × 1 ml of 0.1% (vol/vol) TFA. Samples were eluted with two rounds of 0.5 ml of 50% (vol/vol) ACN.

ROS/MAP frontal cortex samples were processed similarly, except aliquots for the GIS were taken after protein digestion. For MSBB samples, tissue homogenization and protein digestion was performed as described previously^86^. Briefly, samples were homogenized in lysis buffer (50 mM HEPES, pH 8.5, 8 M urea, and 0.5% sodium deoxycholate, 100 ml buffer per 10 mg tissue) with 1x PhosSTOP phosphatase inhibitor cocktail (Sigma-Aldrich). Protein concentration was measured using BCA and confirmed with Coomassie-stained short SDS gels. Aliquots of 100 μg protein were taken from each sample and proteolyzed with Lys-C (Wako, 1:100 w/w) at 21 C for 2 h, then underwent fourfold dilution with 50 mM HEPES. Trypsin (Promega, 1:50 w/w) was then added and digestion continued overnight. The insoluble debris was kept in the lysates for the recovery of insoluble proteins. Samples were acidified to a final concentration of 1% TFA. Samples were centrifuged and the supernatant was desalted with a Sep-Pak C18 cartridge (Waters).

#### Isobaric tandem mass tag (TMT) labeling

Prior to TMT labeling, samples were randomized by age, sex, postmortem interval, cognitive diagnosis, and pathologies into the appropriate number of batches. Randomization was done within each cohort. For ROS/MAP dPFC and Banner samples, peptides from samples and GIS were labeled using the TMT 10-plex kit (ThermoFisher). Two TMT channels were reserved for GIS and the remaining channels were used for individual samples. TMT labeling was performed as described previously^82,87,88^. Briefly, TMT labeling reagents were brought to room temperature. Aliquots of 100 μg of peptide were resuspended in 100 μl of 100 mM TEAB. A total of 256 μl of anhydrous ACN was added to each reagent channel and vortexed gently for 5 min, then, 41 μl of the corresponding TMT channels were transferred to peptide suspensions. Samples were incubated at room temperature for 1 h, then 8 μl of 5% (vol/vol) hydroxylamine was added to quench the reaction. All ten channels were combined and dried by SpeedVac to approximately 150 μl, diluted with 1 ml of 0.1% (vol/vol) TFA, then acidified to a final concentration of 1% (vol/vol) FA and 0.1% (vol/vol) TFA. Samples were desalted with a 200-mg C18 Sep-Pak column (Waters). Each column was first activated with 3 ml of methanol, washed with 3 ml of 50% (vol/vol) acetonitrile, and equilibrated with 2 × 3 ml of 0.1% TFA. The samples were then loaded and each column was washed with 2 × 3 ml of 0.1% (vol/vol) TFA, followed by 2 ml of 1% (vol/vol) FA. Samples were eluted with two rounds of 1.5 ml of 50% (vol/vol) acetonitrile. The ROS/MAP frontal cortex samples were processed similarly, except samples were labeled using the TMT 10-plex kit plus channel 11 (131 C). For MSBB samples, samples and the GIS were labeled with the TMT 11-plex kit (ThermoFisher) according to the manufacturer’s protocol. The GIS was included in channel 126. All 11 channels were mixed equally and desalted with a 100 mg Sep-Pak C18 column (Waters).

#### High pH off-line fractionation

For all samples, fractionation was performed as described previously^24,82,89^. Dried samples were resuspended in high-pH loading buffer (0.07% [vol/vol] NH_4_OH, 0.045% [vol/vol] FA, 2% [vol/vol] ACN). Samples were then loaded onto an Agilent ZORBAX 300Extend-C18 column (2.1 × 150 mm^2^ with 3.5 µm beads), and an Agilent 1100 HPLC system was used for fractionation. Solvent A consisted of 0.0175% (vol/vol) NH_4_OH, 0.01125% (vol/ vol) FA, and 2% (vol/vol) ACN, and solvent B consisted of 0.0175% (vol/vol) NH_4_OH, 0.01125% (vol/vol) FA, and 90% (vol/vol) ACN. The sample elution was performed over a 58.6-min gradient with a flow rate of 0.4 ml/min. The gradient was 100% solvent A for 2 min, from 0% to 12% solvent B over 6 min, from 12 to 40% over 28 min, from 40 to 44% over 4 min, from 44 to 60% over 5 min, and then constant at 60% solvent B for 13.6 min. A total of 96 individual fractions were collected across the gradient, which were then pooled into 24 fractions and dried by SpeedVac.

#### TMT mass spectrometry

Samples were analyzed using liquid chromatography coupled to tandem mass spectrometry (MS2) for ROSMAP frontal cortex, Banner, MSBB, and 90% of ROS/MAP dPFC samples and synchronous precursor selection-based MS3 (SPS-MS3) for 10% of ROS/MAP dPFC samples. For ROS/MAP dPFC, ROS/MAP frontal cortex, and Banner samples, mass spectrometry was performed as described previously^82,90^. Briefly, peptide fractions were resuspended in equal volumes of loading buffer (0.1% FA, 0.03% TFA, 1% ACN). Samples were separated on a self-packed C18 (1.9 μm; Dr. Maisch) fused silica column (25 cm × 75 μm internal diameter; New Objective) by a Dionex UltiMate 3000 RSLCnano liquid chromatography system (ThermoFisher Scientific) and monitored on an Orbitrap Fusion mass spectrometer (ThermoFisher Scientific). For ROS/MAP dPFC and Banner samples, samples were eluted over a 180-min gradient with flow rate at 225 nl/min, and the gradient for buffer B was 3–7% over 5 min, from 7 to 30% over 140 min, from 30 to 60% over 5 min, 60 to 99% over 2 min, constant at 99% for 8 min, then back to 1% for an additional 20 min to equilibrate the column. For ROS/MAP frontal cortex samples, samples were eluted over a 120 min gradient with flow rate of 300 nL/min, and the gradient for buffer B was 1–50%. Buffer A was water with 0.1% (vol/vol) FA, and buffer B was 80% (vol/vol) ACN in water with 0.1% (vol/vol) FA. Acquisition occurred in data-dependent mode using the top speed workflow with a cycle time of 3 s. Each cycle consisted of one full scan (MS1) followed by as many MS2 scans as could fit within the time window. For ROS/MAP dPFC and Banner samples, the full scan was performed with an m/z range of 350–1,500 at 120,000 resolution (at 200 m/z) with automatic gain control set at 4 × 10^5^ and maximum injection time 50 ms, and the most intense ions were selected for higher-energy collision-induced dissociation (HCD) at 38% collision energy with an isolation of 0.7 m/z, resolution of 30,000, automatic gain control of 5 × 10^4^, and maximum injection time of 100 ms. For ROS/MAP frontal cortex samples, MS1 scans were collected at a resolution of 120,000, 400–1400 m/z range, automatic gain control of 4 × 10^5^, maximum injection time of 50 ms. All HCD MS/MS spectra were acquired at a resolution of 60,000, automatic gain control of 5 × 10^4^, isolation width of 1.6 m/z, 35% collision energy, maximum injection time of 50 ms, and dynamic exclusion was set to exclude previously sequenced peaks for 20 s within a 10-ppm isolation window. MS3 runs for ROS/MAP dPFC samples were performed on an Orbitrap Fusion mass spectrometer using the synchronous precursor selection-based MS3 method as described previously^82,88^. For MSBB samples, mass spectrometry was performed as described previously^86,90^. Briefly, samples were run sequentially on a column (75 μm × 20–40 cm, 1.9 μm C18 resin [Dr. Maisch]) with a Q Exactive HF Orbitrap or Fusion mass spectrometer (Thermo Fisher) and eluted using a 2–3 h gradient. The settings for the MS1 scan included m/z range of 410–1600, 60,000 or 120,000 resolution, automatic gain control of 1 × 10^6^, and maximum injection time of 50 ms. Settings for the MS2 scans included fixed first mass of 120 m/z, 60,000 resolution, automatic gain control of 1 × 10^5^, maximum injection time of 100–150 ms, higher-energy C-trap dissociation, normalized collision energy of 35–38%, ~1.0 m/z isolation window with 0.3 m/z offset, and ~15 s dynamic exclusion.

#### Database searches and protein quantification

For ROS/MAP dPFC samples, database searches and protein quantification was performed as described previously^82^. Raw files were analyzed using Proteome Discoverer (v.2.3, ThermoFisher Scientific). MS2 spectra were searched against the canonical UniProtKB human proteome database (downloaded February 2019 with 20,338 total sequences) using the Sequest HT search engine with the following parameters: fully tryptic specificity, maximum of two missed cleavages, minimum peptide length of 6, fixed modifications for TMT tags on lysine residues and peptide N termini (+229.162932 Da) and carbamidomethylation of cysteine residues (+57.02146 Da), variable modifications for oxidation of methionine residues (+15.99492 Da) and deamidation of asparagine and glutamine (+0.984 Da), precursor mass tolerance of 20 ppm. Fragment mass tolerance was set to 0.05 Da for MS2 spectra collected in the Orbitrap and 0.5 Da for the MS2 from the synchronous precursor selection-based MS3 batches. Percolator was used to filter peptide spectral matches and peptides to achieve a FDR of less than 1%. After spectral assignment, peptides were assembled into proteins and filtered once again to a final FDR of 1% based on the combined peptide probabilities. Multi-consensus was employed to achieve parsimony across batches. Shared peptides were assigned to proteins in accordance with the parsimony principle. Reporter ions were quantified from MS2 or MS3 scans using an integration tolerance of 20 ppm with the most confident centroid setting. Banner sample data was also processed as described for the ROS/MAP dPFC samples except that MS2 spectra were searched against the UniProtKB human brain proteome database downloaded in April 2015. Likewise, ROS/MAP frontal cortex and MSBB data were processed similarly as described for Banner data except that MS2 spectra were searched against the UniProtKB human proteome database containing both Swiss-Prot and TrEMBL human reference protein sequences (90,411 target sequences downloaded April 21, 2015) plus 245 contaminant proteins. The top matching protein or “master protein” was defined as the protein with the most unique peptides and the smallest percent peptide coverage.

#### Proteomic data quality control, normalization, and aggregation

We applied the same quality control and normalization procedure to each proteomics dataset separately before they were merged. For each batch, the two GIS were used to check for proteins outside of the 95% confidence interval and set to missing. The MSBB dataset had only one GIS per batch and did not undergo this step. Proteins with missing values in over 50% of samples were also removed. To account for protein loading differences, protein abundance was scaled with sample-specific total protein abundance and log_2_ transformed. Outlier samples were removed using an iterative principal component analysis that identified samples beyond 4 standard deviations from the mean of either the first or second principal component. A linear model was fit to estimate the effects of technical (i.e., MS mode, batch) and demographic factors (i.e., sex, age, post-mortem interval, and final clinical diagnosis of cognitive status) and these effects were regressed from the final estimate of protein abundance. Each dataset was filtered to include only individuals with useable genotyping and standardized using z-scaling before being combined together. For genes with multiple UniProt entries, only the entry with the highest abundance was used. To account for possible hidden confounders, we performed surrogate variable analysis using the SVA package^91^ on the combined proteomic profile and then regressed out 53 hidden factors from the combined proteomic dataset. The final combined proteomic profile after quality control included 722 samples (366 samples from ROS/MAP dPFC, 151 from Banner, 135 from MSBB, and 70 from ROS/MAP frontal cortex) and 9363 proteins.

To examine effects of brain region and case/control definition on the underlying proteomic data, we examined replication rates for pQTL across the individual proteomic datasets. To that end, we performed the pQTL analysis in each proteomic dataset separately. Then, we defined the lead-pQTLs as those that passed Bonferroni-adjusted *p* < 0.05 per gene in the discovery dataset. We next evaluated replication by estimating the π_1_ statistics for the lead pQTLs in the replication dataset using the qvalue package^92^ (Table S17). Additionally, we calculated the Spearman correlations for the pQTL effect sizes in the discovery and replication datasets (Table S17). The high replication rates for pQTLs and high correlations for pQTL effect sizes suggest minimal effects of different brain region and case/control definition in the combined proteomic profile.

### Genotyping

Genotypes were generated from blood or brain-derived DNA using microarrays (ROS/MAP and Banner) and/or WGS (ROS/MAP and MSBB) as described previously^85,93,94^. Initial genotype quality control was performed on each dataset independently. We excluded samples with genotype missing rate >5% and excluded variants meeting any of the following criteria: genotype missing rate >5%, minor allele frequency <5%, Hardy-Weinberg equilibrium *p* value <5 × 10^−7^, and non-biallelic variants. We removed related individuals (i.e. no second-degree or closer relatives) using KING^95^ and removed population outliers using EIGENSTRAT^96^. All participants included in the analysis were of European ancestry. After initial genotype quality control, genotype data were merged, and a second round of population substructure and kinship analysis was applied to verify that the final dataset included only unrelated samples without population outliers.

### Human brain mRNA expression

Expression microarray data from six neurotypical adults was downloaded from the Allen Brain Atlas^48^. The data include multiple probes per gene and multiple samples per brain structure. Z-score normalized expression for probes corresponding to the 13 genes associated with both neurodegenerative and psychiatric traits were downloaded from the Allen Brain Atlas microarray data explorer (https://human.brain-map.org/microarray/search). We arrived at one expression Z-score per gene-brain structure pair by first averaging across probes per gene, then averaging across samples and brains per brain structure.

Human brain single-cell RNA-sequencing data previously generated from the dPFC of 24 cognitively normal donors^50^ were obtained. Then quality control of the data was performed using Seurat (v. 3.1.2)^97^. Genes were retained if they had at least three counts in a cell, and cells were retained if they had unique feature counts between 200 and 2500. Counts were normalized and scaled using the NormalizeData and ScaleData functions.

### Mouse brain single-cell RNA-sequencing

Single-cell RNA sequencing data from mouse cortex and hippocampus described in^98^ were downloaded from the Allen Institute Portal. The dataset encompasses 76,307 single cells. Sequencing results were aligned to exons and introns in the GRCm38.p3 reference genome using STAR, and aggregated intron and exon counts at the gene level were calculated. Matrix files were curated based on genes of interest with Delimit Pro v10/8.1/7. We chose nuclear genes encoding components of the mitochondrial respiratory chain complexes, the mitochondrial ribosome, all the SLC family of transporters plus the 118 genes of interest. Data were assembled with the metadata.csv data using Excel. Data were exported as tab delimited text file and analyzed with the Qlucore Omics Explorer v3.6 to generate 2D t-SNE expression atlases. Data were log2 converted and normalized to a mean of 0 and a variance of 1. 2D t-SNE plots were generated using a perplexity of 40 and default settings^99^.

### Protein-protein interaction

Pairwise protein-protein interactions (PPI) containing only human gene symbols were downloaded from the BioGRID database^51^ (v4.4.179, May 27, 2021). PPIs in BioGRID were curated from primary experimental evidence in the peer-reviewed biomedical literature. We then filtered these PPIs to include only physical protein-protein interactions, which consist of physical association, association, colocalization, and direct interaction.

### Mitochondrial proteins

We used MitoCarta3.0, which is a human curated dataset of 1137 genes^53^. The mitochondrial interactome was generated using high-density human proximity mitochondrial interaction network data^54^, which is composed of 4705 genes (nodes) plus 112,489 interactions (edges). We used Cytoscape 3.7^100^ to generate the mitochondrial interactome.

### Human brain transcriptomes

We used 888 human brain transcriptomes profiled mainly from the dPFC of post-mortem brain samples of 783 donors of European ancestry. Alignment, quality control, and normalization of the data have been described in detail before^22,23,101^. Briefly, RNA-sequencing data was normalized for gene length and GC content. Effects of processing batch, sex, post-mortem interval, RNA integrity, age, cognitive diagnosis, and brain regions were regressed out. After quality control, a total of 13,650 mRNAs remained to be considered for the TWAS.

### Statistical analysis

To estimate pairwise genetic correlations, we performed LD score regression^25^ using the GWAS summary association statistics from participants of European descent. The LD score regression approach is not biased by sample overlap in the GWAS and minimizes the effect of LD structure by restricting the analysis to the well-imputed SNPs in the 1000 Genomes European data^25^. We removed SNPs with extremely large effect sizes (*X*_1_^2^ > 80) since they can unduly influence the regression^25^. False discovery rate (FDR)^102^ was used to correct for multiple testing.

We used the FUSION pipeline^26^ to perform 25 independent PWAS using GWAS results and the reference proteomic and genetic dataset. First, we restricted the genotype data to the SNPs in the LD reference panel provided with the FUSION software, which includes 1,190,321 SNPs from 1000 Genome EUR samples, to minimize the influence of linkage disequilibrium on the analysis. Next, SNP-based heritability for each protein was estimated. Proteins with SNP-based heritability *p* < 0.01 were declared heritable. We found 2909 heritable proteins after removing the proteins in the HLA region (Table S3). Next, for each heritable protein, we estimated the effect of a set of SNPs within a 500Kb window of the gene on its protein abundance, also referred to as the protein “weight”. We applied the BLUP, LASSO, elastic net, and BSLMM prediction models and kept the weights from the best-performing prediction model. Subsequently, we integrated the brain protein weights with each of the 25 GWAS summary statistics to perform 25 independent PWAS. The PWAS Z-score for each gene represents the combined effect of the protein and SNPs on the trait. The PWAS identified the *cis*-regulated proteins associated with the trait. We defined significant proteins as those with FDR-adjusted *p* value < 0.05.

Next, among the significant *cis*-regulated proteins identified in the PWAS above, we performed summary data-based Mendelian Randomization (SMR)^27^ to test whether the brain protein mediates the association between the gene and trait. To do this, we first identified protein quantitative trait loci (pQTL) by testing each SNP within a 500 Kb window of each gene for association with the protein abundance using linear regression. Regression modeling was implemented using PLINK^103^ adjusting for 10 genetic principal components (computed using EIGENSTRAT). Then among the PWAS-significant proteins, we applied SMR to the *cis-*pQTLs and GWAS summary statistics to test if they mediate the association between the gene and trait. Since this mediation can arise from causality, pleiotropy, or linkage disequilibrium, we then used HEIDI^27^ to test for and remove the association likely due to linkage disequilibrium (i.e., HEIDI *p* < 0.05).

Using a Bayesian colocalization approach to complement the SMR/HEIDI method, we performed COLOC^28^ using the marginal association statistics to estimate the posterior probability that a protein and trait share or do not share a genetic variant.

We performed *trans*-pQTL analysis among the independent genome-wide significant SNPs identified from the 25 GWAS. First, genome-wide significant SNPs (*p* value < 5 × 10^−8^ from each GWAS) were selected from autosomes and filtered to the independent SNPs (${r}^{2} \, < \, 0.5$) within 250 kb window using PLINK^103^. Then, we examined the association between each of these SNPs and each brain protein abundance using linear regression as implemented in PLINK, adjusting for 10 genetic PCs. SNPs that were associated with proteins at *p* value < 5 × 10^−8^ and were outside of the 500 kb window of the protein were declared *trans*-pQTLs. Next, we tested whether the proteins regulated by the *trans*-pQTLs mediate the association between the GWAS SNPs and trait using the *trans*-SMR pipeline^27^. To do that, we first performed pQTL analysis for all the SNPs within a 500 kb window of a *trans*-pQTL. Subsequently, we applied *trans*-SMR^27^ on the *trans*-pQTLs by specifying the “–trans” and “–trans-wind 500” options in SMR, which restricted testing to target SNPs and genes located at least 5 Mb apart and defined a window size of 500 kb around the target pQTL SNP for selecting SNPs for the SMR and HEIDI tests. We used a Bonferroni-corrected threshold (0.05 / number of significant pQTLs) to declare a significant *trans*-SMR result.

A *cis*-regulated protein was declared as having evidence consistent with causality or pleiotropy for a trait if it met all of the following A to C criteria: (a) The protein was associated with the trait in the PWAS at FDR *p* < 0.05; (b) The protein showed statistical evidence for mediating the association between SNPs and trait in the SMR test at *p* < 0.05; (c) The association in SMR was not due to linkage disequilibrium as reflected by HEIDI *p* > 0.05; or both of the following D and E criteria: (d) The protein was associated with the trait in the PWAS at FDR *p* < 0.05; (e) The protein and trait have evidence of sharing a genetic variant as reflected by a posterior probability for hypothesis 4 (PP4) > 0.5 in COLOC.

A *trans*-regulated protein was identified as having evidence consistent with causality or pleiotropy if it met all of the following three criteria: (i) its expression was associated with a GWAS-significant SNP in the *trans*-pQTL analysis at *p* value < 5 × 10^−8^; (ii) The protein showed statistical evidence for mediating the association between the GWAS-significant SNP and trait in the SMR test at FDR *p* < 0.05; and (iii) The association in SMR was not due to linkage disequilibrium as reflected by HEIDI *p* > 0.05.

For simplicity, we will refer to proteins consistent with causality or pleiotropy as causal proteins. To examine expression of the causal proteins in different brain regions, we used normalized microarray expression data from the Allan Brain Atlas. The microarray data included multiple probes per gene and multiple samples per brain structure. We arrived at one expression value per gene-brain structure pair by averaging across probes per gene and averaging across samples and brains per brain structure. The Z-score enables comparison of expression for one gene across the different brain regions.

To examine expression of the causal proteins in different brain cell types, we used available human brain single-cell RNA sequencing data generated from the dPFC of 24 cognitively normal donors^50^. For each gene of interest and for each of the five main cell types, we examined whether the gene expression in one particular cell-type differed from its expression in all the other four cell types using the Wilcoxon rank sum test. Significant results were defined as those with Bonferroni-adjusted *p* value < 0.05 (adjusted for all 17,775 genes).

To determine statistical significance for the number of PPIs between the psychiatric causal proteins and the neurodegenerative causal proteins, we used a bootstrap approach. As described above, there were 407 psychiatric and 44 neurodegenerative causal proteins (with 13 proteins shared between the two groups), and 120 physical PPIs between psychiatric and neurodegenerative causal proteins (s_0_ = 120 PPIs). From the BioGRID database (which contains 8942 proteins and 487179 interactions^51^), we randomly selected 404 proteins (because 3 of 407 were not present in BioGRID) and 43 proteins (because 1 of 44 was not present in BioGRID) for the psychiatric and neurodegenerative group, respectively, ensuring that 13 were shared between the two groups. Then we counted the number of physical PPIs between the psychiatric and neurodegenerative causal proteins. This process was repeated 10,000 times and a one-sided empirical *p*-value was calculated as followed: *p* value = ${1+{\sum}_{i=1}^{N}I({s}_{i}\ge {s}_{0})}/{N+1}$, where *N* is the number of iterations, *s*_*i*_ is the count of PPIs between the psychiatric and neurodegenerative causal proteins in iteration *i*, and ${\sum }_{i=1}^{N}I({s}_{i}\ge {s}_{0})$ is the number of bootstrap observations where the count of PPIs, *s*_*i*_, is greater than or equal to *s*_0_.

Gene set enrichment analysis was performed using the python command-line version of GO-Elite (v1.2.5) for human species downloaded in July 2021^104^, which included Biological Process^105^, Molecular Function^105^, Cellular Component^105^, Wiki Pathways^106^, KEGG^105^, CORUM^107^, and REACTOME^108^ databases. Fisher exact test and Z scores were used to test for significant enrichment among the causal proteins of interest using a background of 18215 proteins. Multiple testing was addressed with Benjamini-Hochberg FDR.

To calculate the enrichment of synaptic proteins within the 118 interacting causal proteins, we used the latest and largest synapse proteome database with 7907 unique human proteins^52^. To calculate the enrichment of mitochondrial genes within the 118 proteins, we used 1137 proteins from the MitoCarta3.0 human curated dataset^53^.

Similar analysis pipeline was applied to the 888 human brain transcriptomic data.

### Reporting summary

Further information on research design is available in the Nature Research Reporting Summary linked to this article.

## Supplementary information

Supplementary Information

Description of Additional Supplementary Files

Supplementary Data 1-17

Reporting Summary

## Data Availability

Raw and processed data used in this manuscript are available at https://www.synapse.org/#!Synapse:syn31822992. These data are in whole or in part based on data obtained from the AMP-AD Knowledge Portal (https://adknowledgeportal.org). The AD Knowledge Portal is a platform for accessing data, analyses, and tools generated by the Accelerating Medicines Partnership (AMP-AD) Target Discovery Program and other National Institute on Aging (NIA)-supported programs to enable open-science practices and accelerate translational learning. The data, analyses and tools are shared early in the research cycle without a publication embargo on secondary use. Data is available for general research use according to the following requirements for data access and data attribution (https://adknowledgeportal.org/DataAccess/Instructions). The results of the 25 GWAS were obtained as described in the corresponding references.

## Code availability

We used published pipelines and can provide the code upon request.
