# Supplementary material for: Shared mechanisms across the major psychiatric and neurodegenerative diseases
Source: Nat Commun. 2022 Jul 26;13:4314. doi: 10.1038/s41467-022-31873-5 (PMC9325708; doi:10.1038/s41467-022-31873-5)

Figure S1: Genetic correlation versus percent proteins shared. Among the 12 psychiatric and neurodegenerative traits with at least one causal protein, there were 35 pairs of traits with significant genetic correlation (FDR $p < 0.05$). For these trait pairs, the pairwise genetic correlation is plotted against the percent causal proteins shared by the pair of traits. The percent causal proteins shared was calculated for each trait pair by dividing the number of proteins causal for both traits (i.e., shared causal proteins) by the number of unique proteins causal for either trait. Points are colored by disease category. The best fit line and standard error bounds were determined using the linear regression method implemented in the function geom_smooth() from the R package ‘ggplot’. The trait pair with estimated genetic correlation greater than 1 corresponds to the pair of AD GWAS with different study designs. Spearman rho estimate was performed using a one-tailed test. Shaded grey area surrounding the best-fit line indicates 95% confidence interval.

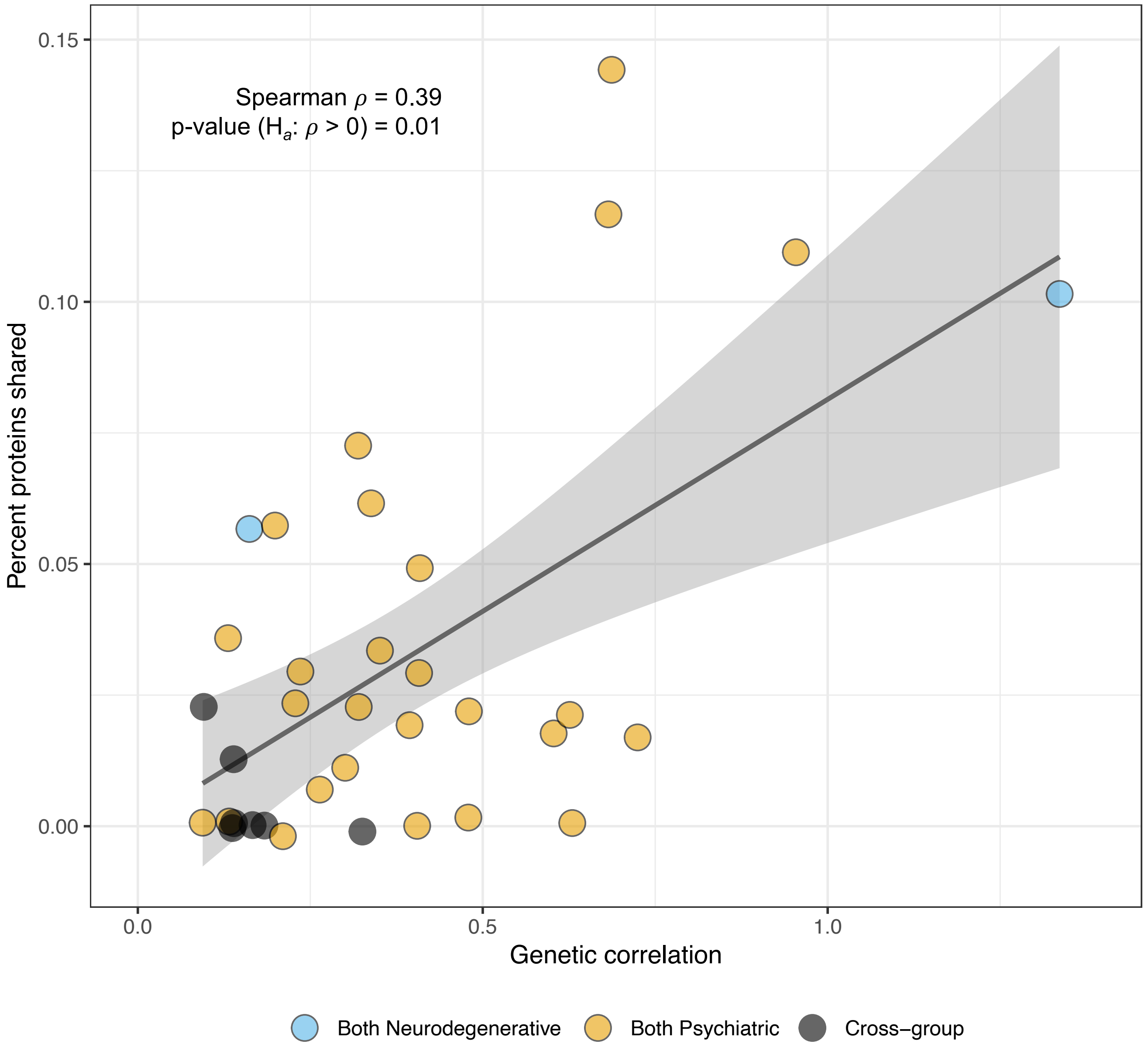

Figure S2: Anatomical expression of the 13 causal proteins shared between the psychiatric and neurodegenerative diseases. **A**. Color was coded for different cell types. **B**. Expression of the 13 shared causal proteins based on single-cell RNA-sequencing data profiled from mouse isocortex and hippocampal formation.

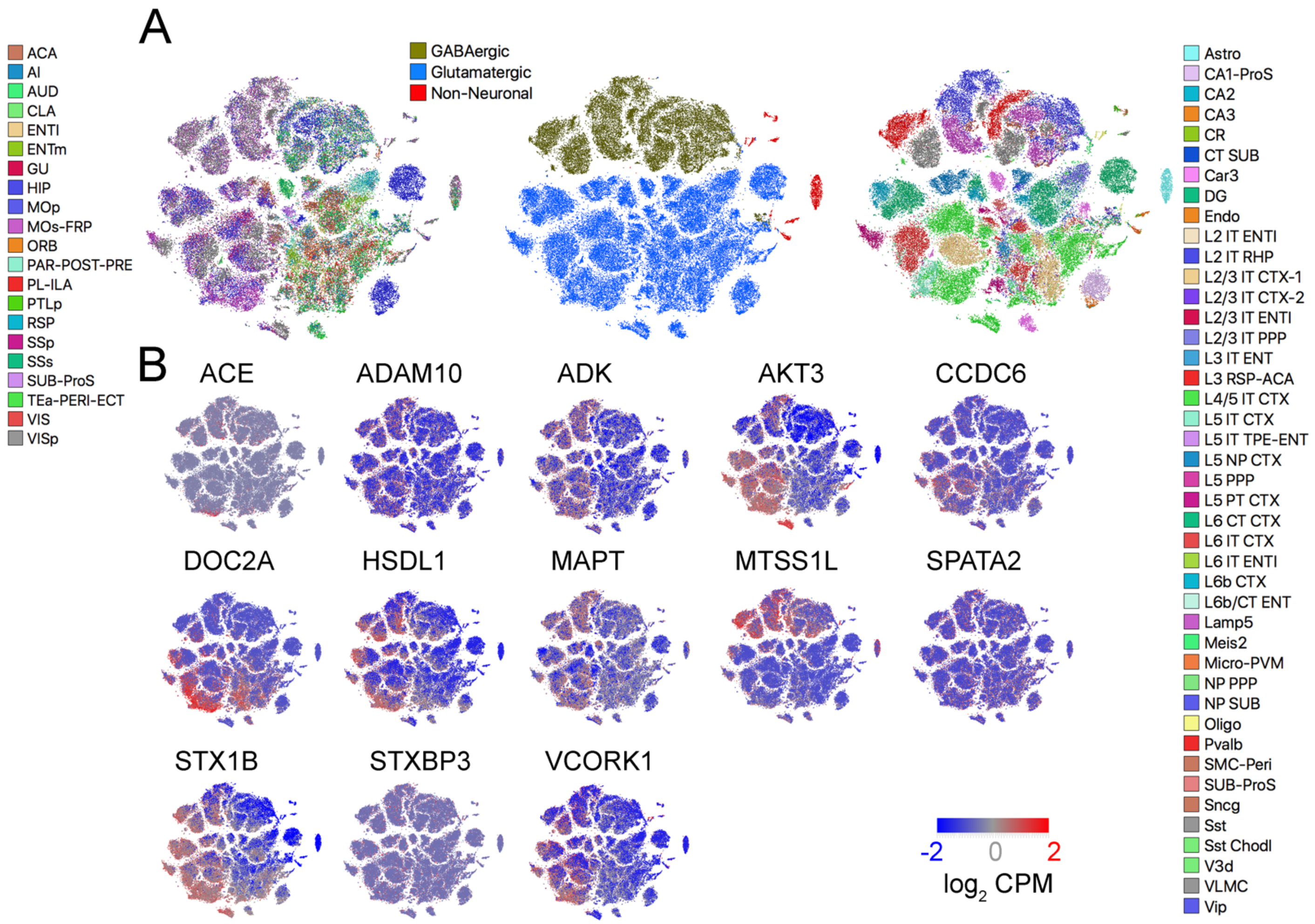

Figure S3: The mitochondrial proximity interactome of the 24 mitochondrial proteins that are part of the 118 interacting psychiatric and neurodegenerative causal proteins. This mitochondrial proximity interactome includes 66 genes.

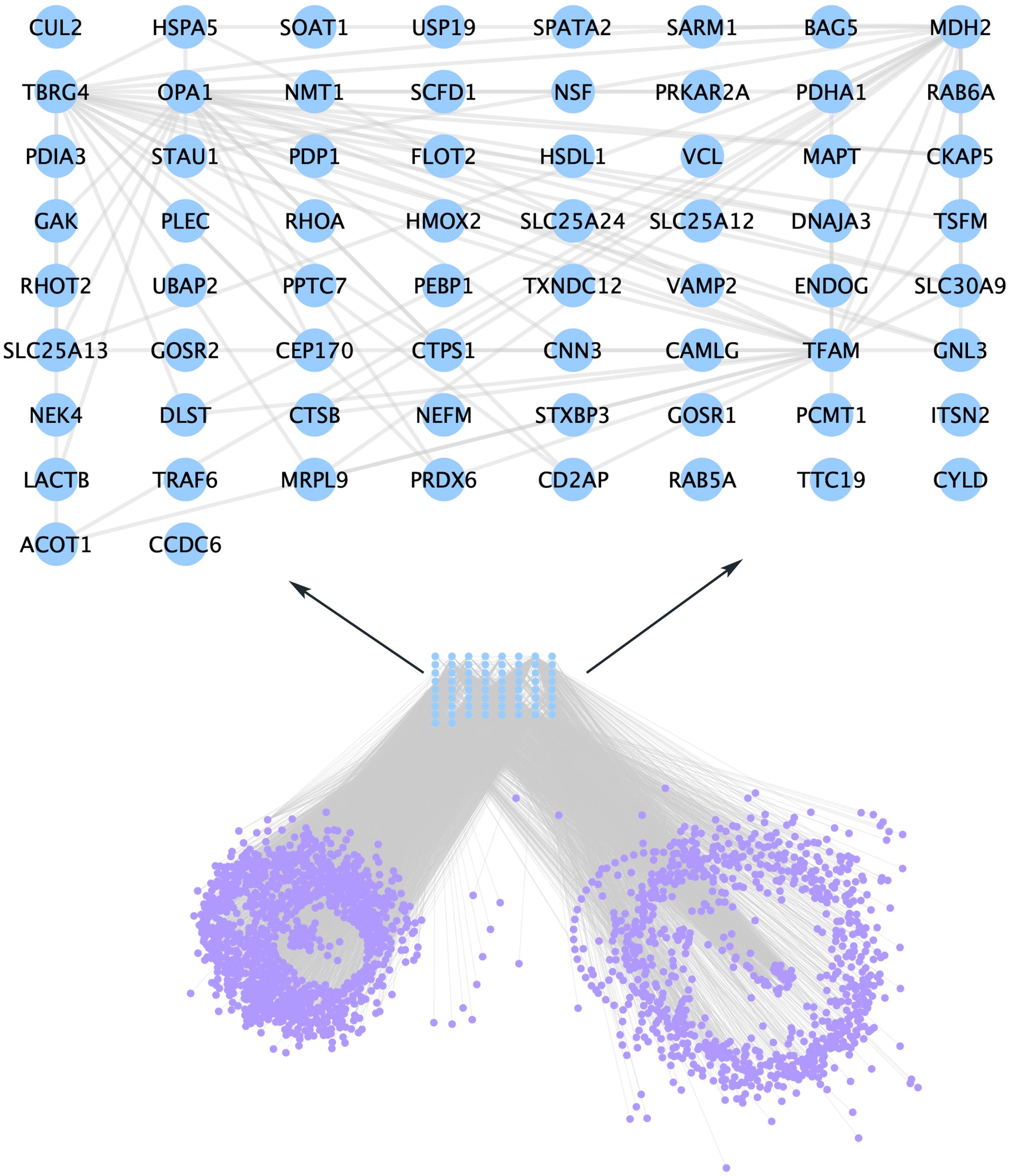

Supplement: Supplementary file 1 — Supplementary Information [file 41467_2022_31873_MOESM1_ESM.pdf]
