# Supplementary material for: Shared mechanisms across the major psychiatric and neurodegenerative diseases
Source: Nat Commun. 2022 Jul 26;13:4314. doi: 10.1038/s41467-022-31873-5 (PMC9325708; doi:10.1038/s41467-022-31873-5)
Supplement: Supplementary file 2 — Description of Additional Supplementary Files [file 41467_2022_31873_MOESM2_ESM.pdf]

Description of Additional Supplementary Files

File Name: Supplementary Data 1
Description: Characteristics of the proteomic datasets.

File Name: Supplementary Data 2
Description: Pairwise genetic correlations based on LD score regression.

File Name: Supplementary Data 3
Description: Heritable proteins tested in the PWAS based on the FUSION pipeline.

File Name: Supplementary Data 4
Description: Proteins consistent with a causal role (cis or trans) and their associated genetic variants based on results of PWAS, SMR/HEIDI, and COLOC for 24 brain traits.

File Name: Supplementary Data 5
Description: Unique causal proteins for each brain trait and shared causal proteins between examined traits.

File Name: Supplementary Data 6
Description: Expression in different brain regions of the 13 causal proteins shared between the psychiatric and neurodegenerative diseases.

File Name: Supplementary Data 7
Description: Expression of all causal proteins in specific cell types.

File Name: Supplementary Data 8
Description: Protein-protein interactions between the neurodegenerative causal proteins and psychiatric causal proteins (118 causal proteins total).

File Name: Supplementary Data 9
Description: Gene set enrichment analysis (GSEA) of the shared and interacting causal proteins.

File Name: Supplementary Data 10
Description: Heritable mRNAs tested in the TWAS using the FUSION pipeline.

File Name: Supplementary Data 11
Description: Genes consistent with a causal role (*cis* or *trans*) and their associated genetic variants based on results of TWAS, SMR/HEIDI, and COLOC for 24 brain traits.

File Name: Supplementary Data 12
Description: Unique causal mRNAs for each brain trait and shared causal mRNAs between examined traits.

File Name: Supplementary Data 13
Description: 24 shared causal mRNAs between the psychiatric and neurodegenerative diseases.

File Name: Supplementary Data 14
Description: Protein-protein interactions between the neurodegenerative causal mRNAs and psychiatric causal mRNAs (145 causal mRNAs total and 171 PPIs).

File Name: Supplementary Data 15
Description: Results of gene set enrichment analysis for the 145 interacting causal mRNAs using Gene Ontology, KEGG, Wiki, REACTOME, and CORUM databases.

File Name: Supplementary Data 16
Description: Overlap between causal proteins and causal mRNAs for each trait, taking into consideration the overlapping genes between genes included in the TWAS and PWAS.

File Name: Supplementary Data 17
Description: Replication rates of pQTLs across the individual proteomic datasets.
