# Supplementary material for: Shared mechanisms across the major psychiatric and neurodegenerative diseases
Source: Nat Commun. 2022 Jul 26;13:4314. doi: 10.1038/s41467-022-31873-5 (PMC9325708; doi:10.1038/s41467-022-31873-5)
Supplement: Supplementary file 4 — Reporting Summary [file 41467_2022_31873_MOESM4_ESM.pdf]

nature portfolio

Corresponding author(s): Aliza Wingo and Thomas Wingo

Last updated by author(s): 2022/06/02

Reporting Summary

Nature Portfolio wishes to improve the reproducibility of the work that we publish. This form provides structure for consistency and transparency in reporting. For further information on Nature Portfolio policies, see our Editorial Policies and the Editorial Policy Checklist.

Statistics

For all statistical analyses, confirm that the following items are present in the figure legend, table legend, main text, or Methods section.

n/a	Confirmed	
☐	☒	The exact sample size (*n*) for each experimental group/condition, given as a discrete number and unit of measurement
☐	☒	A statement on whether measurements were taken from distinct samples or whether the same sample was measured repeatedly
☐	☒	The statistical test(s) used AND whether they are one- or two-sided<br>*Only common tests should be described solely by name; describe more complex techniques in the Methods section.*
☐	☒	A description of all covariates tested
☐	☒	A description of any assumptions or corrections, such as tests of normality and adjustment for multiple comparisons
☐	☒	A full description of the statistical parameters including central tendency (e.g. means) or other basic estimates (e.g. regression coefficient) AND variation (e.g. standard deviation) or associated estimates of uncertainty (e.g. confidence intervals)
☐	☒	For null hypothesis testing, the test statistic (e.g. *F*, *t*, *r*) with confidence intervals, effect sizes, degrees of freedom and *P* value noted<br>*Give P values as exact values whenever suitable.*
☒	☐	For Bayesian analysis, information on the choice of priors and Markov chain Monte Carlo settings
☒	☐	For hierarchical and complex designs, identification of the appropriate level for tests and full reporting of outcomes
☐	☒	Estimates of effect sizes (e.g. Cohen's *d*, Pearson's *r*), indicating how they were calculated

Our web collection on statistics for biologists contains articles on many of the points above.

Software and code

Policy information about availability of computer code

Data collection	An Orbitrap Fusion mass spectrometer (ThermoFischer Scientific) was used to collect raw proteomic files using built-in software.
Data analysis	Proteome Discoverer suite (version 2.3 ThermoFisher Scientific) was used to analyze proteomic raw files. All statistical analyses were performed using R version 3.5.1 (2018-07-02) with the following R packages: ‘dplyr’ version 0.8.0.1, ‘ggplot2’ version 3.1.0, ‘grid’ version 3.5.1, ‘readxl’ version 1.3.0, ‘stringi’ version 1.3.1, ‘ggrepel’ version 0.8.0 , ‘reshape2’ version 3.5.1, and ‘ggcorrplot’ version 0.1.2. Plink version v1.99b was used. FUSION software was downloaded from http://gusevlab.org/projects/fusion. SMR software was downloaded from https://cnsgenomics.com/software/smr. SEURAT V.3.1.2. BioGRID database v4.4.179, May 27,2021. Cytoscape v3.7; KING 2.2.2; Eigenstrat 6.1.4; LDSC 1.0.1; COLOC 5.0.0.9002. qvalue R package version 2.22.0. Excel v16.61.1.

For manuscripts utilizing custom algorithms or software that are central to the research but not yet described in published literature, software must be made available to editors and reviewers. We strongly encourage code deposition in a community repository (e.g. GitHub). See the Nature Portfolio guidelines for submitting code & software for further information.

Data

Policy information about availability of data

All manuscripts must include a data availability statement. This statement should provide the following information, where applicable:

- Accession codes, unique identifiers, or web links for publicly available datasets
- A description of any restrictions on data availability
- For clinical datasets or third party data, please ensure that the statement adheres to our policy

Raw and processed data used in this manuscript are available at https://www.synapse.org/#!Synapse:syn31822992. These data are in whole or in part based on data obtained from the AMP-AD Knowledge Portal (https://adknowledgeportal.org). The AD Knowledge Portal is a platform for accessing data, analyses, and tools

March 2021

generated by the Accelerating Medicines Partnership (AMP-AD) Target Discovery Program and other National Institute on Aging (NIA)-supported programs to enable open-science practices and accelerate translational learning. The data, analyses and tools are shared early in the research cycle without a publication embargo on secondary use. Data is available for general research use according to the following requirements for data access and data attribution (https://adknowledgeportal.org/DataAccess/Instructions). The results of the 25 GWAS were obtained as described in the corresponding references.

Field-specific reporting

Please select the one below that is the best fit for your research. If you are not sure, read the appropriate sections before making your selection.

☒ Life sciences ☐ Behavioural & social sciences ☐ Ecological, evolutionary & environmental sciences

For a reference copy of the document with all sections, see nature.com/documents/nr-reporting-summary-flat.pdf

Life sciences study design

All studies must disclose on these points even when the disclosure is negative.

Sample size	No sample size calculations were used. All available samples with brain proteomic, genetic, and phenotypic data were used for the analysis.
Data exclusions	Outlier samples were removed in the quality control step of proteomic data. This was done through an iterative process of detecting outliers by principal component analysis of the proteomic data and excluding all individuals who were greater than 4 standard deviations from the mean of the first two principal components. Then we included individuals with both proteomic and genome-wide genotyping data for the analyses.
Replication	No replication was performed because we did not have a replication dataset. Furthermore, results from RNA-seq data serve as a source of replication.
Randomization	For proteomic sequencing, samples were randomized by age, sex, PMI, cognitive diagnosis, and pathologies into batches of 8 samples to minimize the batch effects. This is not a clinical trial so randomization is not required.
Blinding	The individuals preparing samples for proteomic sequencing were blinded to phenotypic information.

Reporting for specific materials, systems and methods

We require information from authors about some types of materials, experimental systems and methods used in many studies. Here, indicate whether each material, system or method listed is relevant to your study. If you are not sure if a list item applies to your research, read the appropriate section before selecting a response.

Materials & experimental systems

n/a	Involved in the study	
☒	☐	Antibodies
☒	☐	Eukaryotic cell lines
☒	☐	Palaeontology and archaeology
☒	☐	Animals and other organisms
☐	☒	Human research participants
☒	☐	Clinical data
☒	☐	Dual use research of concern

Methods

n/a	Involved in the study	
☒	☐	ChIP-seq
☒	☐	Flow cytometry
☒	☐	MRI-based neuroimaging

Human research participants

Policy information about studies involving human research participants

Population characteristics	Postmortem brain samples were donated by the ROS/MAP participants (72% female, mean age 90), Banner participants (58% female, mean age 85), and the Mt Sinai Brain Bank donors (64% female, mean age 86).
Recruitment	All research participants signed informed consent and were recruited from their corresponding communities.
Ethics oversight	All the studies (ROS/MAP; Banner, Mt. Sinai Brain Bank) received approval for their studies from an Institutional Review Board at their academic affiliates (Institutional Review Board of Rush University Medical Center; Banner Sun Health Research Institute Institutional Review Board; Mount Sinai/JJ Peters VA Medical Center Brain Bank Institutional Review Board).

Note that full information on the approval of the study protocol must also be provided in the manuscript.
